# Planning adaptive treatment by longitudinal response assessment implementing MR imaging, liquid biopsy and analysis of microenvironment during neoadjuvant treatment of rectal cancer (PRIMO)

**DOI:** 10.1097/MD.0000000000033575

**Published:** 2023-04-28

**Authors:** Georg W. Wurschi, Daniel Güllmar, Nikolaus Gaßler, Joachim Clement, Miriam Kesselmeier, Julia J. Müller-Wurschi, Utz Settmacher, Henning Mothes, Herry Helfritzsch, Yves Liebe, Tobias Franiel, Matthias A. Mäurer, Thomas Ernst, Nils H. Nicolay, Andrea Wittig

**Affiliations:** a Department of Radiotherapy and Radiation Oncology, Jena University Hospital, Friedrich-Schiller University Jena, Jena, Germany; b Clinician Scientist Program, Interdisciplinary Center for Clinical Research (IZKF), Jena University Hospital, Jena, Germany; c Medical Physics Group, Institute of Diagnostic and Interventional Radiology (IDIR), Jena University Hospital, Friedrich Schiller University Jena, Jena, Germany; d Section of Pathology, Institute of Forensic Medicine, Jena University Hospital, Jena, Germany; e Department of Hematology and Medical Oncology, Jena University Hospital, Jena, Germany; f Institute of Medical Statistics, Computer and Data Sciences (IMSID), Jena University Hospital, Friedrich-Schiller University Jena, Jena, Germany; g Center for Clinical Studies, Jena University Hospital, Jena, Germany; h Department of General, Visceral and Vascular Surgery, Jena University Hospital, Jena, Germany; i Department of General, Visceral and Vascular Surgery, Sophien- und Hufeland-Klinikum Weimar, Weimar, Germany; j Department of General, Visceral and Thoracic Surgery, Thuringia-Clinic Saalfeld Georgius Agricola, Saalfeld, Germany; k Department of General and Visceral Surgery, SRH Klinikum Burgenlandkreis Naumburg, Naumburg, Germany; l Institute of Diagnostic and Interventional Radiology (IDIR), Jena University Hospital, Friedrich-Schiller University Jena, Jena, Germany; m University Tumor Center (UTC), Jena University Hospital, Jena, Germany; n Department of Radiation Oncology, University of Leipzig Medical Center, Leipzig, Germany.

**Keywords:** CTC, DWI, MRI, neoadjuvant therapy, rectal cancer, response prediction, TIL

## Abstract

**Introduction:**

Conducting neoadjuvant chemoradiotherapy (CRT) and additional preoperative consolidating chemotherapy (CTx), that is, total neoadjuvant therapy (TNT), improves local control and complete response (CR) rates in locally advanced rectal cancer (LARC), putting the focus on organ preservation concepts. Therefore, assessing response before surgery is crucial. Some LARC patients would either not benefit from intensification by TNT or may reach CR, making resection not mandatory. Treatment of LARC should therefore be based on patient individual risk and response to avoid overtreatment.

The “PRIMO” pilot study aims to determine early response assessment to form a basis for development and validation of a noninvasive response prediction model by a subsequent prospective multicenter trial, which is highly needed for individual, response-driven therapy adaptions.

**Methods:**

PRIMO is a prospective observational cohort study including adult patients with LARC receiving neoadjuvant CRT. At least 4 multiparametric magnetic resonance imaging (MRI) scans (diffusion-weighted imaging [DWI] and hypoxia-sensitive sequences) as well as repeated blood samples in order to analyze circulating tumor cells (CTC) and cell-free tumor DNA (ctDNA) are scheduled. Pelvic radiotherapy (RT, 50.4 Gy) will be performed in combination with a 5-fluorouracil/oxaliplatin regimen in all patients (planned: N = 50), succeeded by consolidation CTx (FOLFOX4) if feasible. Additional (immuno)histochemical markers, such as tumor-infiltrating lymphocytes (TIL) and programmed death ligand 1 (PD-L1) status will be analyzed before and after CRT. Routine resection is scheduled subsequently, nonoperative management is offered alternatively in case of clinical CR (cCR).The primary endpoint is pathological response; secondary endpoints comprise longitudinal changes in MRI as well as in CTCs and TIL. These are evaluated for early response prediction during neoadjuvant therapy, in order to develop a noninvasive response prediction model for subsequent analyses.

**Discussion:**

Early response assessment is the key in differentiating “good” and “bad” responders during neoadjuvant CRT, allowing adaption of subsequent therapies (additional consolidating CTx, organ preservation). This study will contribute in this regard, by advancing MR imaging and substantiating new surrogate markers. Adaptive treatment strategies might build on these results in further studies.

## 1. Introduction

Locally advanced rectal cancer (LARC), comprising Union Internationale Contre Le Cancer stage II and III cancers, has been treated routinely with preoperative (chemo)radiotherapy (RT) and subsequent resection. Optionally, adjuvant chemotherapy (CTx) was advised in case of postoperative risk factors (such as positive resected lymph nodes). This treatment schedule has been standard for over 15 years, resulting in pathological CR (pCR) rates in the range of 10%.^[1]^ Habr-Gama et al hypothesized even before, that patients achieving pCR after preoperative treatment, would have similar long-term outcomes if no resection would be performed.^[2]^ Up to now, several study groups around the world are searching for adequate treatment schedules to establish this “watch and wait” concept in cases of complete response (CR) after neoadjuvant chemoradiotherapy (CRT).

Different approaches have been investigated, favoring an intensification of preoperative treatment by consolidating CTx after initial CRT. Several European trial groups were able to demonstrate increased pCR rates over 50% and increased local tumor control rates by this additional preoperative CTx,^[3–6]^ evolving the so-called “total neoadjuvant therapy” (TNT) regime. This regime, consisting of pelvis RT, for example, with concomitant fluoropyrimidine-based CTx, and followed by consolidating fluoropyrimidine-based CTx, is currently recommended by the German expert associations (Arbeitsgemeinschaft Radiologische Onkologie of DKG [Radiation Oncology working group German Cancer Society] [ARO], Arbeitsgemeinschaft Internistische Onkologie of DKG [Medical Oncology working group of German Cancer Society], Arbeitsgemeinschaft Chirurgische Onkologie of DKG [Surgical Oncology working group German Cancer Society]) for LARC.^[7]^

Based on these promising results, current studies are investigating “*watch and wait*” concepts for those patients responding well to CRT. The recently presented ARO-16 trial was able to demonstrate that 40% of the patients were eligible for resection-free treatment.^[8]^ It hence seems that patients with good tumor regression after surgery could be offered a “*watch and wait*” concept, potentially avoiding resection and increasing quality of life (QoL).

On the other hand, there is a substantial group of patients not reaching pCR after neoadjuvant treatment, so that a resection would still be necessary. Additional preoperative CTx might be considered as ineffective in case of inevitable resection for this group.^[9]^ Deciding if there is an additional benefit when performing preoperative CTx is relevant due to a possible increase of toxicity, like polyneuropathy.^[5]^

Accurate tumor response assessment is the key to allow substantiated decisions whom to offer “*watch and wait*” or not. Even more, early response prediction during neoadjuvant CRT would allow adapting the following treatment, that is, additional CTx to maximize tumor regression in order to obtain pCR versus a straight resection schedule to evade additional toxicity if no pCR is probable. However, differentiating “good responders” and “bad responders” by single clinical examinations only does not seem sufficiently reliable^[10]^ and, to our information, there is no reliable model to anticipate response and benefit from TNT respectively.

Valentini et al^[11]^ were already providing nomograms to estimate tumor control and survival rates based on retrospective analysis. Based on this work, the so-called Neoadjuvant Rectal Score (NAR) was derived, serving as easy-to-apply surrogate for clinical endpoints.^[12,13]^ But to our knowledge, no validated tool based on TNM staging for early response assessment during neoadjuvant treatment has been published up to now.

In this trial, we aim to combine different diagnostic modalities in order to combine them for response assessment in a multimodal cluster. The 3 columns are described subsequently.

One column comprises multiparametric magnetic resonance imaging (MRI), focusing mainly on diffusion-weighted imaging (DWI) and hypoxia-sensitive susceptibility sequences (T2*) in addition to standard T2-weighted images. Both DWI and T2* were established for tumor imaging as they yield information about impaired cell integrity or microenvironment.^[10,14–16]^ There have been promising results published for DWI imaging for rectal cancer diagnosis^[15,16]^; however, there is no reliable option to predict tumor regression by single pre- or post-treatment apparent diffusion coefficient values up to now.^[17]^ Our concept is to establish robust longitudinal assessment in order to extrapolate signal trends, as it is successfully performed with serum tumor markers (carcinoembryonic antigen, CA19-9). T2* on the other hand has recently been investigated in other tumor sites, such as head and neck malignancies.^[18,19]^ At these sites, T2* imaging seems to enable microenvironment evaluation (angiogenesis, hypoxia). However, there is no common use for T2* in rectal cancer. Considering hypoxia is crucial for radiobiological reasons, as hypoxic tissue is one of the key points for radiation resistance. Thus, an association to bad response is probable. Interleukin 6 may serve as surrogate for hypoxia dynamics^[20]^ and will therefore be of interest in this trial.

Tumor environment is also characterized by local tumor-infiltrating lymphocytes (TILs). For example, these are approved as independent prognostic factor in breast cancer.^[21,22]^ But currently there is no standard use in rectal cancer.^[23]^ Consequently, we aim to analyze pre- and post-treatment specimens of TILs and surface antigens (CD3, CD4, CD8, and CD45) and to correlate treatment-related changes with tumor regression as second column.

Further longitudinal trends will serve as third column. We plan to perform liquid biopsies longitudinally in order to detect therapy-induced changes of circulating tumor cells (CTCs) and cell-free tumor DNA (ctDNA). Both are proofed as surrogate markers in other malignancies, but their role in response assessment of rectal cancer remains still unclear.^[24–26]^ Longitudinal trends could serve as surrogate for therapy response.^[25]^ Additionally, we focus on treatment-related changes of surface proteins (e.g., programmed death ligand 1 [PD-L1]). Detection of CTCs or ctDNA may be investigated postoperatively for its significance in relapse diagnostics.^[24]^

The PRIMO trial serves as pilot trial to hypothesize a noninvasive response prediction model, which will be tested and validated in a larger subsequent multicenter study. Secondly, the earliest time point for response estimation should be identified and be then validated in the subsequent trial, in order to allow differentiation between “good responders” and “bad responders” as early as possible during neoadjuvant therapy.

## 2. Material and methods

### 2.1. Design

**Figure 1.**
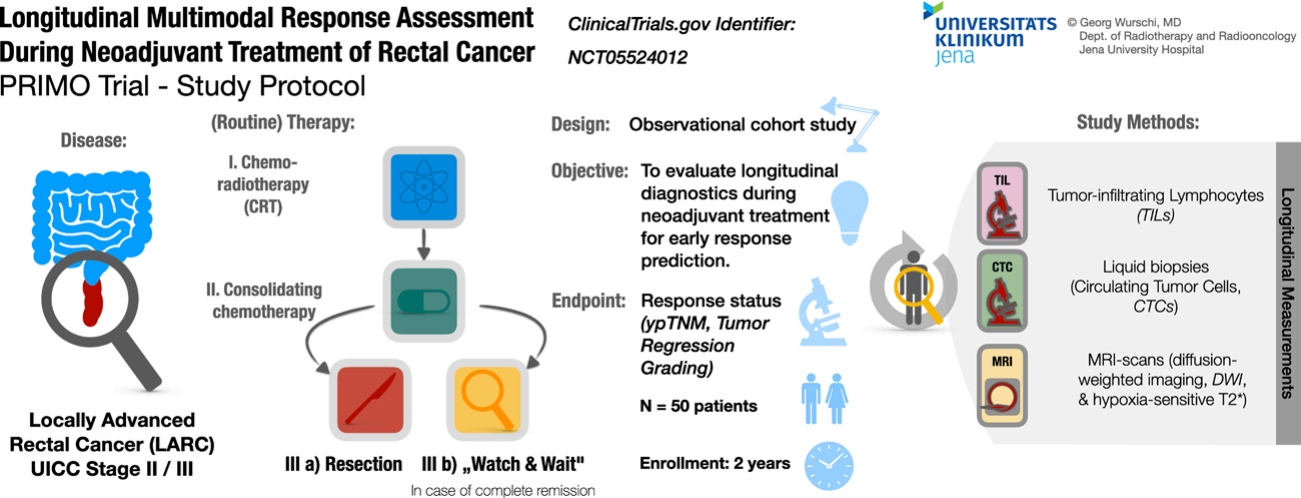
A graphical abstract, summarizing the trial design and scheduled interventions of the PRIMO trial: In this observational pilot study, we aim to analyze N = 50 patients with Locally Advanced Rectal Cancer (LARC) by longitudinal diagnostics in order to develop early noninvasive response assessment models.

This trial, entitled “*Planning Adaptive Treatment by Longitudinal Response Assessment Implementing MR Imaging, Liquid Biopsy and Analysis of Microenvironment during Neoadjuvant Treatment of Rectal Cancer*” (*PRIMO*), is a monocentric observational pilot cohort study (Fig. 1) to be conducted at Jena University Hospital (Jena, Germany). The trial protocol (current version 1.2, 2023-03-01) is structured on the basis of the *Standard Protocol Items: Recommendations for Interventional Trials* guidelines^[27]^ and conforms to the international *Consolidated Standards of Reporting Trials* guidelines.^[28]^ It is additionally provided in this work (Supplemental Digital Content 1, http://links.lww.com/MD/I827). The implementation and reporting of the trial follows the principles of *Good Clinical Practice*^[29]^; this trial was registered at clinicaltrials.gov (ID: NCT05524012) prior to recruitment.

Patients with LARC, defined as Union Internationale Contre Le Cancer Stage II/III cancer, are eligible for this trial. We aim to enroll N = 50 patients (Table 1). These patients are subjected to a neoadjuvant CRT, which is international standard of care. Each patient will be followed-up for 5 years.

**Table 1:** Inclusion criteria for the PRIMO trial.

Inclusion criteria	
LARC, UICC stage II/III	Each T4 tumor; Each T3 tumor when located distally (0–6 cm) T3 and location >6 cm: in case of risk factors (e.g., N+, EMVI+, MSP+, >cT3b)
Sufficient liver/renal function; No severe cytopenia	GFR > 45 mL/min; liver enzymes < 2.5 ULN Neutrophils ≥ 3 × 10^9^/l; Thrombocytes ≥ 100 × 10^9^/l; Hemoglobin ≥ 6 mmol/l
No contraindications for MRI	Incompatible endoprosthesis/cardiac devices; severe claustrophobia; severe adipositas (BMI > 30 kg/m^2^)
No further relevant secondary diagnosis	No homozygous DPD-deficiency; No severe cardiac disease No severe pulmonary disease No severe psychologic disorder For female patients: No pregnancy. No history of preceding chemo(radio)therapy or parallel participation in other clinical trials.

BMI = body mass index, EMVI = extramural vascular invasion, GFR = glomerular filtration rate, MRI = magnetic resonance imaging, MSP+ = infiltration of Mesorectal Plane, N+ = positive lymphnodes, UICC = *Union Internationale Contre Le Cancer*, ULN = upper limit of normal range.

Pretreatment tumor specimens and resection or biopsy specimens after neoadjuvant treatment will be appraised by 1 single expert pathologist (NG) in order to classify the tumor regression grading (TRG), according to Werner/Hoefler,^[30,31]^ and to perform secondary assessments of TILs and their surface antigen cluster (CD3+, CD4+, CD8+, CD45+). By pre- and post-treatment tumor grading, the established NAR by George et al^[12,32]^ will be calculated for secondary comparison. Furthermore, pre- and post-treatment expression of surface proteins (like PD-L1) will be compared. We require 5 serial biopsies if nonoperative management is performed.

**Figure 2.**
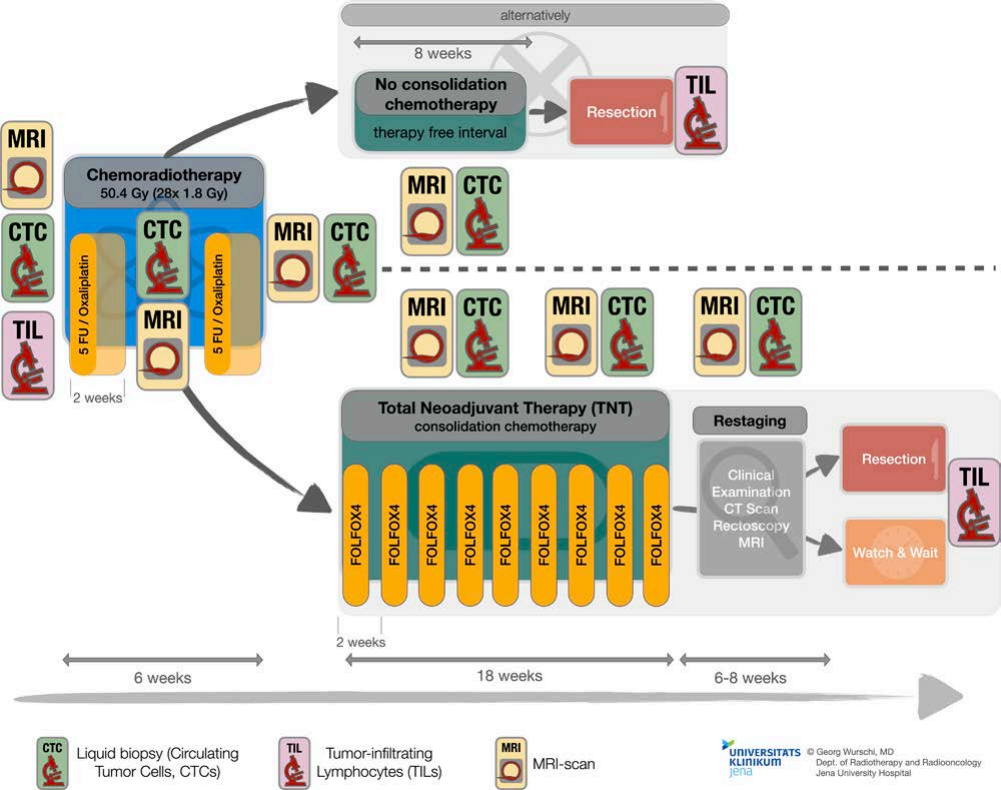
Workflow during the PRIMO trial until primary endpoint: All patients are receiving chemoradiotherapy, followed by either consolidation chemotherapy (total neoadjuvant therapy, TNT) or an 8-wk therapy free interval. During this 2-phase therapy, repeated MRI scans and liquid biopsies (circulating tumor cells, CTCs) are performed. A baseline of histology and tumor-infiltrating lymphocytes (TILs) is compared with resection or biopsy specimens after therapy. Overall duration is 34 wk (complete TNT) or 16 wk (no consolidation chemotherapy), respectively. MRI = magnetic resonance imaging.

Multimodal longitudinal assessments are scheduled during neoadjuvant treatment. These comprise especially multiparametric MRI scans of the rectum to quantify tumor regression, including noncontrast enhanced T2-weighted imaging as well as additional DWI- and hypoxia-sensitive T2*-sequences. Four to 6 MRI scans are scheduled, depending if “TNT” would be indicated (Fig. 2). Radiological features of all sequences are obtained longitudinally from 3 regions of interest within the tumor area as well as a region of interest placed in unaffected rectum within the radiation field for comparison purposes. Each will be positioned consent-based by 2 radiotherapists and, additionally, by a semiautomatic, cluster-based approach.

Liquid biopsies, comprising CTCs and ctDNA, will be performed from routine blood samples regularly during neoadjuvant therapy and follow-up in addition to routine blood samples. These comprise C-reactive protein, interleukin 6 and carcinoembryonic antigen as well as standard blood counts, liver enzymes and creatinine. ctDNA will be investigated for somatic mutations, such as KRAS p.G12D/p.G12V/p.G13D or BRAF V600E, by sequencing. CTC analyses will be performed by fluorescence microscopy, focusing on EpCAM and further antigen detection (e.g., PD-L1). Measurement of the CTCs and ctDNA is planned regularly during preoperative treatment as well as annually during follow-up for 5 years.

QoL will be repeatedly measured applying EORTC QLQ-C30 v3.0^[33,34]^ and CR-29^[35]^ questionnaires during neoadjuvant treatment and follow-up. A detailed overview of the assessments and study procedures according to the *Standard Protocol Items: Recommendations for Interventional Trials* guideline is attached as Supplemental Digital Content 2, http://links.lww.com/MD/I828.

The REDCap electronic data capture tools^[36,37]^ will be used for data collection and management, ensuring compliance to local data privacy restrictions and promoting data quality. A professional clinical trial manager (JMW) manages trial conduction and will perform data curation and quality checks.

### 2.2. Treatment description

Informed consent of each patient is required for study participation. CRT is then conducted in a 2-phase design (Fig. 2). It consists of standard pelvis RT (50.4 Gy) and, if necessary, inguinal RT (45.0 Gy; involved groin nodes or distal tumor infiltration) in combination with 2 cycles of concomitant CTx (5-fluorouracil, plus oxaliplatin, once every 2 weeks - q2w).

In the second phase, additional consolidating CTx (9 cycles FOLFOX4, q2w) is advised for all patients in case of additional risk factors (as specified in Table 1: positive lymph nodes, infiltration of the mesorectal plane or extramural vessel invasion, distal tumor location or T4 tumors) and good tolerability of primary neoadjuvant CRT, according to current therapy recommendations of the German colorectal tumor expert associations (ARO, *Arbeitsgemeinschaft Internistische Onkologie* of DKG [Medical Oncology working group of German Cancer Society], *Arbeitsgemeinschaft Chirurgische Onkologie* of DKG [Surgical Oncology working group German Cancer Society]).^[7]^ This regime is also known as “Total Neoadjuvant Therapy” (TNT). Dose reduction of scheduled CTx in case of toxicity and adverse events, such as hemotoxicity and neuropathy, will be realized following the prescribing information. Depending on restaging results, resection is performed after 34 weeks or patients are offered a “*watch and wait*” approach in case of CR and good compliance.

In case of high toxicity (>CTC° III) or noncompliance during simultaneous CRT, a rectum resection is scheduled 8 weeks after completion of RT (week 16). Performing this 2-phase design allows adaption to individual patient consent and tolerability during ongoing therapy. Simultaneously, it ensures adequate treatment intensity even if no additional CTx would be realizable, as this group is still receiving a neoadjuvant therapy analogous to the approved ARO-04-protocol.^[1]^ We are conducting this 2-phased design (Fig. 2) for over 2 years as local standard at our institution.

### 2.3. Endpoints

The primary endpoint is defined as the tumor regression after neoadjuvant CRT and will be evaluated at week 16 or 34 respectively, depending on consolidating CTx. It will be quantified by a TRG according to Werner/Hoefler^[30]^ in resection or biopsy specimens, depending if nonoperative management was conducted. TRG 1a/b or 2 is considered as response (see Table 2). CR will be evaluated as secondary endpoint and is defined as a TRG 1a/b. Further secondary endpoints (Table 3) comprise correlation of radiological features to TRG and NAR^[12,32]^ as well as the correlation of longitudinal changes of CTCs/ctDNA and TILs to TRG, respectively. Therapy-related side effects (according to EORTC-CTCAE) and QoL (based on QLQ-C30 and CR-29 questionnaires) are secondary objectives, as well as 5-years progression free survival and overall survival after 5 years. Relapses occurring within the follow-up period serve as endpoint for ctDNA and CTC analyses regarding early diagnosis.

**Table 2:** Tumor regression grading (TRG) according to Werner and Höfler^[30]^ and Becker et al^[31]^ TRG 1a/b is considered as complete response (cR).

Tumor regression grading according to Becker et al		
1a	Complete response (CR)	No vital tumor cells detectable
1b	Subtotal response (SR)	<10% vital tumor cells
2	Partial response (PR)	10%–50% vital tumor cells
3	Minimale response (MR)	>50% vital tumor cells
	No response (NR)	No histological evidence of regression

**Table 3:** Primary and secondary endpoints of the PRIMO trial.

Endpoints	
Primary endpoint	Response rate according to tumor regression grading (TRG)-score/ypTNM
Secondary endpoints	Longitudinal changes in MRI during neoadjuvant treatment
	Presence/longitudinal phenotypic changes of ctDNA and CTC
	Presence/phenotypic changes of tumor-infiltrating lymphocytes (TILs)
	Surrogates of inflammation (CrP, IL-6) and tumor markers (CEA)
	Treatment-related toxicity (EORTC-CTCAE v5.0)
	Overall survival (OS), progression free survival (PFS), recurrence rate
	Quality of life (QoL) according to EORTC QLQ-C30 and QLQ-CR29

Tiered analyzes is planned, evaluating endpoints referencing to neoadjuvant treatment when enrolled patients have met the primary endpoint and analyzing secondary endpoints referring to follow-up results after 5 years follow-up.

CEA = carcinoembryonic antigen, CRP = C-reactive protein, CTC = circulating tumor cell, ctDNA = cell-free tumor DNA, IL-6 = interleukin 6, MRI = magnetic resonance imaging.

### 2.4. Statistics

The primary hypothesis of the primary endpoint is about the predictive value of multimodal diagnostics to predict response in LARC patients who underwent neoadjuvant therapy. Secondary hypothesis are related to longitudinal changes in these parameters to identify the earliest time point at which response assessment is valid.

Depending on which treatment regime was successfully administered, we consider 2 groups of patients. Group I comprises patients receiving complete TNT (8–9 courses FOLFOX; group I). Group II comprises patients without consolidating CTx (group II). Note, the primary hypothesis is analyzed in group I.

The scope of this pilot study is the estimation (i.e., descriptive statistics) and not the statistical testing. The sample size is motivated by the feasibility, that is, by the number of eligible patients during the recruitment time of 2 years. Based on preceding case series at our institution, 50 eligible LARC patients are expected to be enrolled and, among them, at least 35 are expected to receive complete consolidating CTx. Overall, we expect at least 30 of 50 (i.e., 60%) patients to show response (TRG 1a/b/2) in histological examination after neoadjuvant treatment. Assuming a statistical power of 0.80 and applying a 2-sided significance level of 0.05, an area under the receiver operating characteristic curve of about 0.76 could be detected in a sample of 35 patients and of 0.72 in a sample of 50 patients (see also Supplemental Digital Content Figure 3, http://links.lww.com/MD/I829 for a detailed overview of the expected area under receiver operating characteristic curve depending on sample size and response rate).

Depending on the variable level of measurement and distribution, appropriate statistical tests will be performed. Missing variables will be excluded from analyses if necessary or compensated by adequate statistical tests for repeated analyses (e.g., Generalized Estimating Equations). Reasons for missing data will be reported. Sensitivity analyses are planned, where appropriate. See also the Supplemental Digital Content, Document 1, http://links.lww.com/MD/I827, which provides the original study protocol, for a more detailed description of the statistical analyses. An academic statistician (MK) will support the analyses.

### 2.5. Planned timeline

Enrollment of this trial started in Q4 2022 (first patient in, first visit) and is still ongoing. A 2-years-enrollment period is planned until Q4 2024, so that it is anticipated to reach the primary endpoint in Q3 2025. Analysis of the primary endpoint will be performed then; a tiered analysis of the secondary endpoints is planned at the end of the 5-years follow-up (last patient out, last visit; Q3 2030).

## 3. Discussion

The PRIMO trial aims to evaluate different multimodal parameters in the context of longitudinal response assessment in order to provide a multimodal cluster of diagnostics to identify “good responders” as well as “bad responders” as soon as possible during neoadjuvant treatment. There is an unmet need for such early noninvasive response assessment, as the benefit of additional consolidating CTx for individual patients needs to be estimated early during CRT to aid the decision when to offer TNT and when not in order to avoid either over- or undertreatment. Currently, appropriate patient selection prior to treatment is debated and needs further development.^[9]^

There are promising results for the different diagnostic modalities regarding response assessment. However, to our knowledge, repeated longitudinal evaluation and such combination of modalities has not been combined to address response assessment before. Especially repeated liquid biopsy seems to be a promising approach with only little available data for its significance regarding LARC.

As there is no similar multimodal prediction model reported up to now, this investigational pilot study is required for analyzing the feasibility of this approach and to design a prospective multicenter trial in a second step. Therefore, we prefer analyzing the investigated modalities descriptively for their significance of longitudinal response prediction in a single-center cohort, but the planned sample size limits statistical significance on the other hand. We aim to minimize these effects by implemented data quality controls and predefined imaging protocols or analyzing procedures (further specified in the original trial protocol, see Supplemental Digital Content 1, http://links.lww.com/MD/I827).

If we would succeed to design a longitudinal model for response anticipation and validate it in a subsequent multicenter trial, such a model may contribute to response-driven and individual risk-based therapy adaptions, maximizing patient benefit.

## Acknowledgments

We would like to thank Professor Emmanouil Fokas for his kind support and supervision of this project during *ARO Mentoring Day 2022 (Berlin*).

## Author contributions

**Conceptualization:** Georg W. Wurschi, Daniel Güllmar, Nikolaus Gaßler, Joachim Clement, Julia J. Müller-Wurschi, Matthias A. Mäurer, Andrea Wittig.

**Data curation:** Julia J. Müller-Wurschi.

**Formal analysis:** Miriam Kesselmeier.

**Funding acquisition:** Georg W. Wurschi.

**Investigation:** Georg W. Wurschi, Daniel Güllmar, Nikolaus Gaßler, Joachim Clement, Utz Settmacher, Henning Mothes, Herry Helfritzsch, Yves Liebe, Tobias Franiel.

**Methodology:** Georg W. Wurschi, Miriam Kesselmeier.

**Project administration:** Georg W. Wurschi, Julia J. Müller-Wurschi.

**Resources:** Daniel Güllmar, Nikolaus Gaßler, Joachim Clement, Utz Settmacher, Henning Mothes, Herry Helfritzsch, Yves Liebe, Tobias Franiel, Thomas Ernst.

**Supervision:** Nils H. Nicolay, Andrea Wittig.

**Visualization:** Georg W. Wurschi.

**Writing – original draft:** Georg W. Wurschi.

**Writing – review & editing:** Miriam Kesselmeier, Thomas Ernst, Nils H. Nicolay, Andrea Wittig.

## Supplementary Material






