# Supplementary material for: Planning adaptive treatment by longitudinal response assessment implementing MR imaging, liquid biopsy and analysis of microenvironment during neoadjuvant treatment of rectal cancer (PRIMO)
Source: Medicine (Baltimore). 2023 Apr 28;102(17):e33575. doi: 10.1097/MD.0000000000033575 (PMC10146036; doi:10.1097/MD.0000000000033575)
Supplement: Supplementary file 1 [file medi-102-e33575-s001.pdf]

# Studienprotokoll

## Adaptive Therapieplanung durch longitudinale MR-Bildgebung, liquid biopsy und Analyse des Microenvironment zur Beurteilung des Therapieansprechens während neoadjuvanter Radiochemotherapie des Rektumkarzinoms (PRIMO)

*Planning adaptive treatment by longitudinal response assessment implementing MR imaging, liquid biopsy and analysis of microenvironment during neoadjuvant treatment of rectal cancer (PRIMO)*

| | |
|---|---|
| Studienakronym: | PRIMO |
| Protokollversion: | Finalversion 1.2 vom 01.03.2023 |
| Vertraulichkeitshinweis: | Der Inhalt vom vorliegenden Studienprotokoll ist vertraulich zu behandeln und darf ohne Zustimmung der Studienleitung weder mündlich noch schriftlich an Unbeteiligte weitergegeben werden. |

# Inhaltsverzeichnis


Inhaltsverzeichnis ..... 2
1. Allgemeine Informationen ..... 4
1.1 Beteiligte Personen, Institutionen und Gremien ..... 4
1.2 Unterschriften ..... 5
1.3 Zusammenfassung ..... 6
1.4 Synopse ..... 7
1.5 Ablaufdiagramm ..... 11
1.6 Visitenplan ..... 12
1.7 Verzeichnis der Abkürzungen ..... 14
2. Fragestellung bzw. Hintergrund ..... 15
2.1 Therapie des Rektumkarzinoms ..... 15
2.1.1 Etablierte Therapiekonzepte und deren Weiterentwicklung ..... 15
2.1.2 Rolle der MR-Bildgebung in der neoadjuvanten Therapie ..... 16
2.2 Fragestellung und Begründung des Vorhabens ..... 16
2.3 Begründung der Behandlungs- und Untersuchungsverfahren ..... 17
3. Studienziele und Endpunkte ..... 18
3.1 Primäres Studienziel und primärer Endpunkt ..... 18
3.2 Sekundäre Studienziele und sekundäre Endpunkte ..... 18
4. Studiendesign und -beschreibung ..... 19
4.1 Art der Studie ..... 19
4.2 Art der Therapiezuordnung ..... 19
4.3 Indikationen zur TNT ..... 19
4.4 Zahl und Art der Vergleichsgruppen ..... 19
4.5 Patientenrekrutierung / Umfang der Studie ..... 19
4.6 Zeitplan ..... 20
5. Studienpopulation ..... 21
6. Studienablauf ..... 23
6.1 Screening und Patientenidentifikationsliste ..... 23
6.2 Aufklärung und Einwilligung ..... 23
6.3 Studienverlauf ..... 23
6.4 Studienbedingte Maßnahmen ..... 24
6.5 Laborkontrollen und Dosisreduktion der TNT ..... 26
6.6 Studienende ..... 27
6.7 Vorgehen nach dem (vorzeitigen) Ausscheiden ..... 27
7. Unerwünschte Ereignisse ..... 29
8. Biometrie ..... 30

8.1 Endpunkte ........ 30
8.2 Definition von Auswertungskollektiven ........ 30
8.3 Planung des Studienumfanges (Fallzahlplanung) ........ 30
8.4 Zwischenauswertung(en) ........ 31
8.5 Statistische Analyse ........ 31
8.6 Präsentation der Ergebnisse ........ 32
9. Datenmanagement ........ 33
9.1 Patientenidentifikationsliste ........ 33
9.2 Liste der Verantwortlichkeiten ........ 33
9.3 Datenerhebung/Datenverarbeitung ........ 33
9.4 Aufbewahrung der Studienunterlagen (Archivierung) ........ 33
9.5 Datenschutz ........ 34
10. Ethische Belange und administrative Regelungen ........ 35
10.1 Deklaration von Helsinki und Gute klinische Praxis ........ 35
10.2 Ethik-Kommissionen ........ 35
10.3 Nachträgliche Änderungen ........ 35
10.4 Registrierung ........ 35
10.5 Finanzierung ........ 35
10.6 Abschlussbericht und Publikation ........ 35
11. Anlagen ........ 36
11.1 Anlage 1 Regressions-Skala (TRG) nach TU München ........ 36
11.2 Anlage 2: Lebensqualitätfragebogen EORTC QLQ-C30 ........ 37
11.3 Anlage 3: Lebensqualitätfragebogen EORTC QLQ-CR29 ........ 39
11.4 Anlage 4: Fragebogen zur Erfassung therapiebedingter Nebenwirkungen ........ 41
11.5 Anlage 5: Karnofsky Performance Status (KPS) ........ 43
12. Literatur ........ 44

# 1. Allgemeine Informationen

## 1.1 Beteiligte Personen, Institutionen und Gremien

| | |
|---|---|
| **Studienleiterin**<br><br>Name: Prof. Dr. Andrea Wittig-Sauerwein<br>Adresse: Universitätsklinikum Jena, Klinik für Strahlentherapie und Radioonkologie<br>Bachstraße 18, 07745 Jena<br>Tel.: 03641-9-328401<br>E-Mail: andrea.wittig-sauerwein@med.uni-jena.de | **Biometrikerin (beratend)**<br><br>Name: Dr. Miriam Kesselmeier<br>Adresse: Universitätsklinikum Jena, Institut für Medizinische Statistik, Informatik und Datenwissenschaften<br>Bachstraße 18, 07745 Jena<br>Tel.: 03641-9-396627<br>E-Mail: miriam.kesselmeier@med.uni-jena.de |
| **Stellvertreter der Studienleiterin**<br>**Koordinator**<br><br>Name: Dr. Georg Wurschi<br>Adresse: Universitätsklinikum Jena, Klinik für Strahlentherapie und Radioonkologie<br>Bachstraße 18, 07745 Jena<br>Tel.: 03641-9-328416<br>E-Mail: georg.wurschi@med.uni-jena.de | **Kooperationspartner / Labor**<br><br>Name: Dr. rer. biol. hum. Joachim Clement<br>Adresse: Universitätsklinikum Jena, Klinik Innere Medizin II, Abteilung Hämatologie und Internistische Onkologie<br>Am Klinikum 1, 07747 Jena<br>Tel.: 03641-9-325820<br>E-Mail: joachim.clement@med.uni-jena.de |
| **Kooperationspartner**<br><br>Name: Dr. Daniel Güllmar<br>Adresse: Universitätsklinikum Jena, Institut für diagnostische und Interventionelle Radiologie (IDIR), AG Medizinische Physik,<br>Philosophenweg 3, 07743 Jena<br>Telefon: 03641-9-390705<br>E-Mail: daniel.guellmar@med.uni-jena.de | **Kooperationspartner / Referenzinstitut**<br><br>Name: Prof. Dr. Nikolaus Gaßler<br>Adresse: Universitätsklinikum Jena, Institut für Pathologie,<br>Am Klinikum 1, 07747 Jena<br>Telefon: 03641-9-397001<br>E-Mail: nikolaus.gassler@med.uni-jena.de |
| **Kooperationspartner**<br><br>Name: Prof. Dr. Utz Settmacher<br>Adresse: Universitätsklinikum Jena, Klinik für Allgemein-, Viszeral- und Gefäßchirurgie<br>Am Klinikum 1, 07747 Jena<br>Telefon: 03641-9-322601<br>E-Mail: avg@med.uni-jena.de | **Kooperationspartner**<br><br>Name: Dr. Herry Helfritzsch<br>Thüringenkliniken Saalfeld, Klinik für Allgemein-, Viszeral- und Thoraxchirurgie<br>Rainweg 68, 07318 Saalfeld<br>Telefon: 03671-541300<br>E-Mail: bauchchirurgie@thueringen-kliniken.de |
| **Kooperationspartner**<br><br>Name: PD Dr. Henning Mothes<br>Sophien- und Hufelandklinikum Weimar, Klinik für Allgemein-, Viszeral und Gefäßchirurgie<br>Henry-van-de-Velde-Straße 2, 99425 Weimar<br>Telefon: 03643-573100<br>E-Mail: avc@klinikum-weimar.de | **Kooperationspartner**<br><br>Name: Prof. Dr. Yves Liebe<br>SRH Klinikum Naumburg, Klinik für Allgemein und Viszeralchirurgie<br>Humboldtstraße 31, 06618 Naumburg<br>Telefon: 03445-210-200-1<br>E-Mail: allgemeinchirurgie-nmb@klinikum-burgenlandkreis.de |

## 1.2 Unterschriften

---

*Prof. Dr. Andrea Wittig-Sauerwein*, Studienleiterin Datum

---

*Dr. Georg Wurschi*, Stellvertreter Datum

---

*Dr. Miriam Kesselmeier*, Biometrikerin (beratend) Datum

## 1.3 Zusammenfassung

In einer prospektiven Pilotstudie soll der Vorhersagewert multimodaler Diagnostik für das Therapieansprechen des Rektumkarzinoms während der neoadjuvanten Radiochemotherapie (RChT) geprüft werden. Ein Teil der Patienten erhält nach simultaner RChT eine zusätzliche konsolidierende Chemotherapie vor der Operation im Sinne einer „total neoadjuvanten Therapie“ (TNT). Dieses multimodale Behandlungskonzept wird derzeit bei lokal fortgeschrittenen Rektumkarzinomen zur Maximierung der Tumorregression empfohlen. Hieraus ergeben sich die Fragestellungen, ob (1) die neoadjuvante RChT zukünftig an das individuelle Tumoransprechen angepasst werden sollte, (2) welche Patientengruppe von dem intensivierten neoadjuvanten Therapiekonzept profitieren wird und (3) ob bei kompletter Tumorremission ein Verzicht auf eine Operation vertretbar ist.

Für die Umsetzung wäre die Etablierung zuverlässiger Vorhersageparameter für das Tumoransprechen nötig. Mit diesem Ziel werden therapieinduzierte Veränderungen im Verlauf einer routinemäßigen neoadjuvanten RChT mittels multiparametrischer MRT-Sequenzen, insbesondere der DWI-MRT (engl. *Diffusion-weighted magnet resoncance tomography)*, geprüft. Das Resektat wird hinsichtlich der makroskopischen und mikroskopischen Tumorregression standardisiert untersucht und diese Ergebnisse auf einen Zusammenhang mit den MR-Signalveränderungen analysiert. Als unabhängige Prognosemarker werden lokal tumorinfiltrierende Lymphozyten (TILs) und im Blut zirkulierende Tumorzellen (CTCs, *engl. circulating tumor cells)* longitudinal im Therapieverlauf untersucht. Zudem werden die Lokalrezidivrate, Fernmetastasierungsrate, Therapieverträglichkeit und Lebensqualität erfasst. Die Pilotstudie soll die Grundlage zur Definition von validen, multimodalen Surrogatmarkern für das Tumoransprechen während der neoadjuvanten RChT legen.

## 1.4 Synopse

| | |
|---|---|
| **Titel der Studie (Deutsch)** | Adaptive Therapieplanung durch longitudinale MR-Bildgebung, liquid biopsy und Analyse des microenvironment zur Beurteilung des Therapieansprechens während neoadjuvanter Radiochemotherapie des Rektumkarzinoms |
| **Titel der Studie (Englisch)** | Planning adaptive treatment by longitudinal response assessment implementing MR imaging, liquid biopsy and analysis of microenvironment during neoadjuvant treatment of rectal cancer |
| **Kurzbezeichnung der Studie (Akronym)** | PRIMO |
| **Studienleiter** | Prof. Dr. med. Andrea Wittig-Sauerwein, Universitätsklinikum Jena, Klinik für Strahlentherapie und Radioonkologie |
| **Stellvertreter des Studienleiters; Koordinator** | Dr. med. Georg Wurschi, Universitätsklinikum Jena, Klinik für Strahlentherapie und Radioonkologie |
| **Indikation/Zielpopulation/ Erkrankung** | Patienten mit fortgeschrittenem Rektumkarzinom (ab UICC Stadium II) im Rahmen der neoadjuvanten Radiochemotherapie |
| **Studiendesign/Methodik** | Offene, prospektive, nicht-randomisierte, monozentrische Pilotstudie (Studie nach Berufsordnung der Ärzte §15) |
| **Ziele der klinischen Prüfung/Zielstellung** | **Primäres Ziel:** Prädiktiver Wert longitudinaler MR-Parameter für das histologische Therapieansprechen auf eine neoadjuvante 5-Fluorouracil (5-FU)-basierte Radiochemotherapie (RChT) +/- konsolidierende Chemotherapie (ChT) mit FOLFOX4 als total neoadjuvante Therapie (TNT).<br><br>**Sekundäre Ziele:** Erfassung der Volumenregredienz des Tumors, der Expression von Oberflächenproteinen des Tumors (z.B. PD-L1) sowie des Microenvironments (z.B. Hypoxie, Zellintegrität) unter Therapie, Identifikation des frühestmöglichen Nachweises eines Tumoransprechens anhand von MR-Parametern im Therapieverlauf;<br>Veränderung der Anzahl von tumorinfiltrierenden Lymphozyten (TILs) sowie Veränderungen des Phänotyps (z.B. exprimierte Oberflächenproteine) im Therapieverlauf;<br>Anzahl, Phänotyp (insbesondere exprimierte Oberflächenproteine) zirkulierender Tumorzellen (CTCs) und zirkulierender Tumor-DNA (ctDNA) im Therapieverlauf;<br>Identifikation von strahlentherapiebedingten Veränderungen im tumorfreien Rektum;<br>sowie Erfassung der therapiebedingten Nebenwirkungen nach CTCAE, Lebensqualität nach EORTC QLQ-C30, Lokalrezidivrate nach 5 Jahren, Rate an Fernmetastasen nach 5 Jahren und Überleben nach 5 Jahren |
| **Zielgrößen/-kriterien/ Endpunkte** | **Primäre Zielgröße/Hauptzielkriterium:** Ansprechen auf TNT (binär)<br><br>**Sekundäre Zielgrößen/Nebenzielkriterien**:<br>• Komplettremission bzw. Vorhandensein und Volumen eines Resttumors nach neoadjuvanter RChT +/- anschließender konsolidierender ChT („TNT“-Konzept), ypTNM und TRG<br>• Veränderungen des Phänotyps der Tumorzellen nach neoadjuvanter RChT, insbesondere exprimierter Oberflächenproteine<br>• Longitudinale multiparametrische MR-Signalveränderungen<br>• MR-Signalveränderungen in einer tumorfreien *region of interest* des Rektums als Referenzstruktur |

| | |
|---|---|
| | • Analyse von TILs und deren Phänotyp (insb. exprimierte Oberflächenproteine) in der prätherapeutischen Probe sowie im Resektat der Operation<br>• Longitudinale Veränderungen der Zahl und des Phänotyps (insb. exprimierte Oberflächenproteine) der CTCs und in der ctDNA<br>• Therapiebedingte Nebenwirkungen nach CTCAE<br>• Lebensqualität nach EORTC QLQ-C30 / CR-29 im Therapieverlauf und Follow up<br>• *Progression free* survival (PFS) sowie Therapieversagen *(„treatment failure“)* i.S. von Lokalrezidiv oder Fernmetastasen<br>• 5 Jahres Überlebensrate *(„overall survival“,* OS) |
| **Anzahl Studienteilnehmer** | Die Fallzahl leitet sich aus der Machbarkeit im vorgegebenen Zeitrahmen ab.<br><br>Erwartet werden 50 Patienten, die eine 5 Fluorouracil (FU)-basierte neoadjuvante Radiochemotherapie (RChT) erhalten und in die Studie eingeschlossen werden.<br><br>Es wird erwartet, dass bei mindestens 15 der 50 eingeschlossenen Patienten die Durchführung einer kompletten konsolidierenden Chemotherapie mit 9 Zyklen FOLFOX4 möglich ist (Analysegruppe I). Entsprechend wird von 35 Patienten ausgegangen, die keine vollständige konsolidierende Chemotherapie erhalten können (Analysegruppe II).<br><br>Bei einer angenommenen vollständigen Ansprechrate (pCR) von mindestens 20% in der Analysegruppe I (≥3 / 15 Patienten) kann eine Fläche unter der ROC-Kurve (*engl. receiver operating characteristic*; AUC) von 0,94 mit einer Power von 0,8 bei einem Signifikanzniveau von 0,05 nachgewiesen werden. |
| **Einschlusskriterien** | - histologisch bestätigtes Adenokarzinom entsprechend der Einschlusskriterien des unteren (0-6 cm ab ano) oder mittleren Rektumdrittels (6-12 cm ab ano) [1]:<br>  o mindestens uT3, cN0 der cN+, cM0 (UICC Stadium II bis III)<br>  o jedes cT3 bei tiefem Sitz<br>  o bei T3 mit Tumor im mittleren Rektumdrittel bei Vorliegen mindestens einer der zusätzlichen Faktoren: befallene perirektale Lymphknoten (N+); Infiltration des mesorektalen Fettgewebes >5mm (cT3c/d) bzw. der mesorektalen Faszie (MSP+); extramurale Gefäßinvasion (EMVI+)<br>  o jedes T4<br>  o bei Sitz im oberen Rektumdrittel nur bei besonderen Risikofaktoren für eine zu erwartende R1-Situation oder ausgeprägtem Lymphknotenbefall: T4, MSP+ oder N2 (entsprechend der Empfehlung der interdisziplinären Tumorkonferenz)<br>  o bei Sitz im oberen Rektumdrittel nur bei besonderen Risikofaktoren für eine zu erwartende R1-Situation: T4, MSP+ (entsprechend der Empfehlung der interdisziplinären Tumorkonferenz)<br>- Indikation zur neoadjuvanten Radiochemotherapie sowie ggf. anschließender konsolidierender Chemotherapie als total neoadjuvante Therapie (TNT) vor operativer Resektion<br>- Alter ≥ 18 Jahre<br>- Karnofsky Performance Status (KPS) 70-100% |

[1] gemessen in der starren Rektoskopie

| | |
|---|---|
| | - Keine vorhergehende Strahlentherapie des Beckens<br>- Keine vorangegangene Chemotherapie<br>- Keine weitere aktive Tumorerkrankung.<br>    o ausgenommen nicht-invasive Basaliome oder nicht-invasive Plattenepithelkarzinome der Haut, therapierte Prostata-Karzinome bei PSA < 0,2 ng/ml<br>- Keine schwere Begleiterkrankung:<br>    o Keine schwere obstruktive oder restriktive Lungenerkrankung<br>    o Keine schwere allergische Erkrankung, die eine kontinuierliche Medikation erfordert<br>    o Keine schwere Herzerkrankung (kongestive Herzinsuffizienz, Angina pectoris, neue koronare Herzkrankheit, unkontrollierte Herzrhythmusstörungen oder Leitungsstörungen)<br>    o Keine schwere Magen-Darm-Erkrankung, wie aktive peptische Ulkuskrankheit<br>    o Keine schwere Beeinträchtigung der Leberfunktion (Bilirubin, Transaminasen, alkalische Phosphatase >2,5-Fache der oberen Grenze des Normalbereichs)<br>    o Keine schwere Beeinträchtigung der Nierenfunktion mit einer glomerulären Filtrationsrate (GFR) <30 ml/min,<br>    o Keine unkontrollierte endokrine Erkrankung<br>    o Keine vorbestehende schwere psychische oder schwere organische Hirnerkrankung (z. B. vorbestehende Epilepsie)<br>- Keine Kontraindikation gegen eine Therapie mit Oxaliplatin oder 5-FU. Folgende hämatologische Werte dürfen nicht unterschritten werden:<br>    o Neutrophile ≥3 x $10^9$/l<br>    o Thrombozyten ≥100 x $10^9$/l<br>    o Hämoglobin ≥6 mmol/l<br>- vollständiges Staging vor Radiochemotherapie, bestehend aus DRU, Rektoskopie/Koloskopie mit ERUS, MRT Becken (nativ), CT Thorax / Abdomen / Becken mit Kontrastmittel (alternativ, sofern indiziert PET-CT)<br>- Ausschluss einer homozygoten DPD-Exon-Skipping-Mutation [1]<br>- Keine psychologischen, familiären, soziologischen oder geografischen Umstände, die die Einhaltung des Studienprotokolls und des Zeitplans für die Nachbeobachtung behindern könnten; diese Umstände sollten mit dem Patienten vor der Registrierung für die Studie besprochen werden.<br>- Frauen dürfen nicht schwanger sein oder stillen. Frauen und Männer im gebärfähigen Alter, die in die Studie aufgenommen werden, werden darüber aufgeklärt, dass die Behandlung teratogen sein kann. Patienten und Patientinnen müssen zustimmen, während der Radiochemotherapie und Chemotherapie sichere Maßnahmen zur Verhinderung einer Empfängnis zu ergreifen.<br>- Vor der Patientenregistrierung muss eine schriftliche Einwilligung des Patienten/der Patientin nach Aufklärung gemäß ICH/GCP erteilt sein.<br>- Keine parallele Teilnahme an einer anderen interventionellen klinischen Prüfung<br>- Keine Kontraindikation für MRT:<br>    o Keine Herzschrittmacherimplantation<br>    o Keine Hüft-total-Endoprothese (Hüft-TEP)<br>    o Keine Metallimplantate von >15 cm Länge<br>    o Kein nicht MR-gängiges Port-System<br>    o Keine ausgeprägte Adipositas (BMI >30 $kg/m^2$) |
| **Behandlungen/Verfahren**<br><br>*Vgl. mit Visitenplan, Tabelle* | **Screening:** -42-0 Tage vor Einschluss; Erfüllung Einschlusskriterien<br><br>**V1:** Studieneinschluss, Baseline-Diagnostik: d0-d14; *Forschungs-MRT + CTCs; Lebensqualität nach EORTC QLQ-C30 / CR-29* |

| | |
|---|---|
| *1* | **V2-V5:** Kombinierte Radiochemotherapie (50,4 Gy, 28 Fraktionen; simultan 2 Zyklen 5-FU + Oxaliplatin); d15-56; *Forschungs-MRT + CTCs; anschließend therapiebedingte Nebenwirkungen nach CTCAE-Kriterien und Lebensqualität nach EORTC QLQ-C30 / CR-29*<br><br>**V6[2]-V15[3]**: konsolidierende Chemotherapie („TNT", 9 Zyklen FOLFOX4): d64-d224; *Forschungs-MRT + CTCs*<br><br>Anschließend **Restaging** und **OP (Resektat für primären Endpunkt) /** optional **„watch & wait" (serielle Biopsie für primären Endpunkt):** d232-238[2]; *TRG + TILs; CTCs (post-operativ)*<br><br>Alternativ, bei Kontraindikationen zur TNT: Restaging, dann **OP** (d106-112) nach 8 Wochen Therapie-freiem Intervall[3]: *TRG + TILs; CTCs (post-operativ)*<br><br>**V16-V22:** Nachsorgen (Woche 42 – 274[2] / Woche 24 - 256[3]): *CTCs + Therapiebedingte Nebenwirkungen nach CTCAE-Kriterien und Lebensqualität nach EORTC QLQ-C30 / CR-29* |
| **Zeitplan (Studiendauer)** | <u>patientenbezogen</u>: 9 Monate bei vollständiger konsolidierender Chemotherapie bis zum Erreichen des primären Endpunktes, 5 Monate bei unvollständiger konsolidierender Chemotherapie bis zum Erreichen des primären Endpunktes. Jeweils 5 Jahre Nachbeobachtung ab dem Zeitpunkt des Therapieendes.<br><br><u>studienbezogen:</u><br>Rekrutierungsdauer: ca. 24 Monate<br>Einschluss erster Patient: Q4/2022<br>Einschluss letzter Patient: Q4/2024<br>Letztes Follow-up: Q3/2030<br>Gesamtdauer: 8 Jahre<br><br><u>Auswertung:</u> Das Tumoransprechen wird histologisch am Operationspräparat nach Tumorresektion abschließend bewertet (Erreichen primärer Endpunkt), die Auswertung folgt daher in Q3/2025.<br>Finale Auswertung: Q4/2030 |
| **Prüfzentren** | geplant: n = 1 |
| **Statistische Methoden** | Primärer Endpunkt: deskriptive Beschreibung der Verläufe und der Veränderung der MRT-Parameter; Untersuchung des prädiktiven Potenzials der MRT-Parameter hinsichtlich des Ansprechens auf die neoadjuvante Radiochemotherapie +/-TNT mittels der AUC.<br><br>Sekundäre Endpunkte: Verwendung adäquater Methoden der beschreibenden und schließenden Statistik |
| **Finanzierung** | - Sachmittel im Rahmen des IZKF *„clinician scientist"*-Programms<br>- Drittmittel im Rahmen des *„LIFE Talent Funds"* (FSU Jena)<br>- Hausmittel für weitere Aufwendungen |

[2] bei konsolidierender Chemotherapie („TNT", Analysegruppe I)
[3] ohne zusätzliche konsolidierende Chemotherapie (direkte Operation, Analysegruppe II)

## 1.5 Ablaufdiagramm

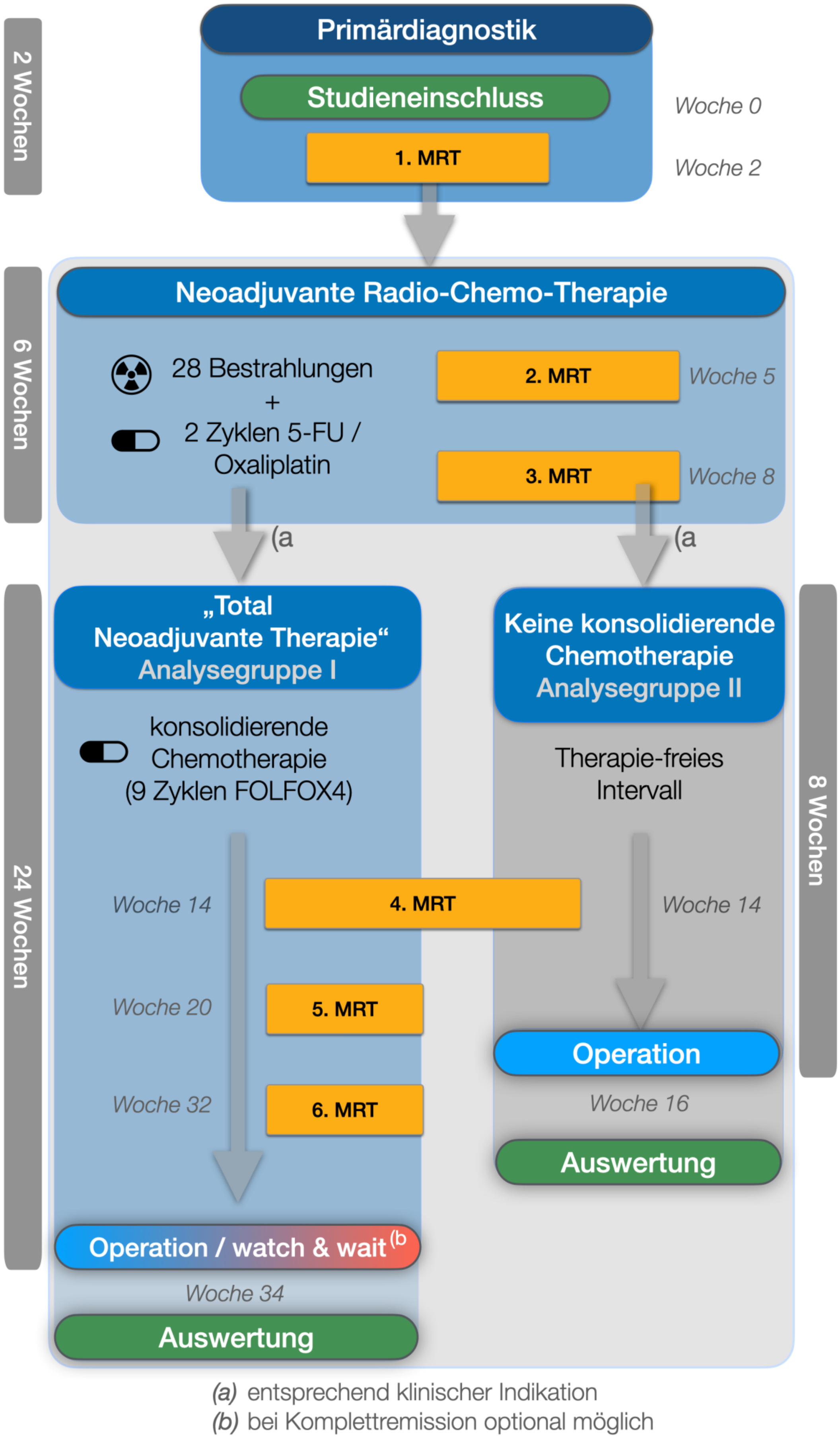


***Abbildung 1** Ablaufdiagramm mit Übersicht über die studienspezifischen MRTs. Patienten, die eine vollständige konsolidierende Chemotherapie erhalten (Analysegruppe I), werden mit sechs MRTs untersucht und nach 34 Wochen operiert bzw. im „watch and wait“-Konzept beobachtet. Patienten, bei denen keine konsolidierende Chemotherapie durchgeführt wird, erhalten vier MRTs und werden nach 16 Wochen operiert.*

## 1.6 Visitenplan

Tabelle 1: *Aufführung der Visiten und der stattfindenden (studienspezifischen) Ereignisse; Kennzeichnung der Visiten (V) sowie des Operationszeitpunktes (OP)*

| *Erhebungszeitpunkt* | **Screening** | **V0** | **V1** | **V2[3]** | **V3** | **V4** | **V5** | **V6** | **V7** | **V8** | **V9 / OP[1]** | **V10[2]** | **V11[2]** | **V12[2]** | **V13[2]** | **V14[2]** | **V15[2]** | **OP[2]** | **Auswertung primärer Endpunkt** |
|---|---|---|---|---|---|---|---|---|---|---|---|---|---|---|---|---|---|---|---|
| **Tage** (relativ zum Studieneinschluss) | -42-0 | 1 | 2-14 | 15-28 | 29-35 | 36-49 | 50-56 | 64-70 | 78-84 | 92-98 | 106-112 | 120-126 | 134-140 | 141-147 | 162-168 | 176-182 | 218-224 | 232-238 | |
| **Wochen** (relativ zum Studieneinschluss) | **-6-0** | **1** | **2-3** | **3-4** | **5** | **6-7** | **8** | **10** | **12** | **14** | **16** | **18** | **20** | **22** | **24** | **26** | **32** | **34** | |
| | **Studienablauf** | | | | | | | | | | | | | | | | | | |
| 5-FU-/Oxaliplatin Therapie (2 Zyklen, 14d) | | | | X | | X | | | | | | | | | | | | | |
| Radiatio (28 Fraktionen) | | | | X | X | X | X | | | | | | | | | | | | |
| TNT: FOLFOX4 Chemotherapie (9 Zyklen, 14d) | | | | | | | | X[2] | X[2] | X[2] | X[2] | X[2] | X[2] | X[2] | X[2] | X[2] | | | |
| Probenexzision bei Diagnosestellung (TNM) | X | | | | | | | | | | | | | | | | | | |
| Rektumexstirpation / serielle Biopsie[2] (ypTNM / TRG-Score) | | | | | | | | | | | X[1] | | | | | | | X[2] | |
| | **Einschluss** | | | | | | | | | | | | | | | | | | |
| Aufklärung / Einwilligung | | X | | | | | | | | | | | | | | | | | |
| Einschlusskriterien | | X | | | | | | | | | | | | | | | | | |
| Demographische Daten | | X | | | | | | | | | | | | | | | | | |
| | **Apparative Untersuchungen** | | | | | | | | | | | | | | | | | | |
| **Forschungs-MRT** | | | X | | X | | X | | | X | | | X[2] | | | | X[2] | | |
| **Bestimmung TILs** | X | | | | | | | | | | | X[1] | | | | | | X[2] | |
| **Bestimmung CTCs** | | | X | | X | | X | | | X[1]/ X[2] | X[1,5] | | | | | | X[2] | X[2,5] | |
| | **Krankheitsverlauf/-daten** | | | | | | | | | | | | | | | | | | |
| Lebensqualität (QLQ-C30, CR-29) | | X | | | | | X | | | | | | | | | | | | |
| Toxizität (CTCAE-Bogen) | | X | | | X | | X | | | X | | | X[2] | | | | X[2] | | |
| KPS (%) | | X | | X | X | X | X | X | X | X | X[1] / X[2] | X[2] | X[2] | X[2] | X[1] / X[2] | X[2] | | | |
| Bestimmung CEA | | X | | | X | | X[1] / X[2] | | | X[1] / X[2] | | | X[2] | | | | X[2] | | |
| Bestimmung IL-6 | | X | | | X | | X[1] / X[2] | | | X[1] / X[2] | | | X[2] | | | | X[2] | | |
| Laborwerte (Leukozyten, Thrombozyten, GFR) | X | | | X | X | X | | X[2] | X[2] | X[2] | X[2] | X[2] | X[2] | X[2] | X[2] | X[2] | X[2] | | |

*1 – Nur für Patienten ohne vollständige konsolidierende RChT*

*2 – Nur für Patienten mit vollständiger konsolidierender RChT (9 Zyklen FOLFOX4)*

| *Erhebungszeitpunkt* | **V16** | **V17** | **V18** | **V19** | **V20** | **V21** | **V22** | **Studienabschluss** |
|---|---|---|---|---|---|---|---|---|
| **Monate** (relativ zum Studieneinschluss) | 6[1] / 10,5[2] | 10[1] / 14,5[2] | 16[1] / 20,5[2] | 28[1] / 32,5[2] | 40[1] / 44,5[2] | 52[1] / 56,5[2] | 64[1] / 68,5[2] | |
| **Wochen** (relativ zum Studieneinschluss) | **24[1] / 42[2] (8 W. post-OP)** | **40[1] / 58[2] (6 Monate post-OP)** | **64[1] / 82[2] (12 Monate post-OP)** | **112[1] / 130[2] (24 Monate post-OP)** | **160[1] / 178[2] (36 Monate post-OP)** | **208[1] / 226[2] (48 Monate post-OP)** | **256[1] / 274[2] (60 Monate post-OP)** | |
| | **Studienablauf** | | | | | | | |
| **Nachsorge** | X | X | X | X | X | X | X | |
| | **Apparative Untersuchungen** | | | | | | | |
| **Forschungs-MRT** | | | | | | | | |
| **Bestimmung TILs** | | | | | | | | |
| **Bestimmung CTCs** | X | X | X | X | X | X | X | |
| | **Krankheitsverlauf** | | | | | | | |
| **Tumorspezifische Nachsorge** | X | X | X | X | X | X | X | |
| Lebensqualität (QLQ-C30, CR-29) | X | | X | | | | X | |
| Therapiebedingte Nebenwirkungen (CTCAE-Bogen) | X | X | X | X | X | X | X | |
| Bestimmung CEA | X | X | X | X | X | X | X | |
| Laborwerte (Leukozyten, Thrombozyten, GFR) | X | X | X | X | X | X | X | |

*3 – In der Regel erfolgt der Beginn der RChT innerhalb von 14 Tagen nach V0. Kommt es hierbei zu Verzögerungen, so wird der gesamte Studienablauf um die Zeit der Verzögerung verschoben. Die Abstände zwischen den nachfolgenden Maßnahmen bleiben davon unberührt.*

*4 – bei unvollständiger konsolidierender RChT[1] (Analysegruppe II) entfallen die Visiten V10 – V15*

*5 – post-operative Bestimmung der CTCs (OP + 0-5d)*

## 1.7 Verzeichnis der Abkürzungen

| | |
|---|---|
| 5-FU | 5-Fluorouracil |
| ADC | *apparent diffusion coefficient,* (scheinbarer Diffusionskoeffizient) |
| AK | Ausschlusskriterien |
| AE | *Adverse event* (unerwünschtes Ereignis) |
| AUC | *Area under the curve (Fläche unter der ROC-Kurve)* |
| CEA | Carcinoembryonales Antigen (Tumormarker zur Verlaufsbeurteilung) |
| CTCAE | *Common toxicity criteria adverse events* (standardisierte Einschätzung von Nebenwirkungen entsprechend der EORTC) |
| CTCs | *Circulating tumor cells* (zirkulierende Tumorzellen) |
| ctDNA | *circulating tumor DNA* (zirkulierende Tumor-DNA) |
| cR | *complete remission* (komplette Remission) |
| CT | Computertomografie |
| DW-MRI | *Diffusion-weighted magnet resoncance tomography* (diffusionsgewichtete MRT) |
| DWI | *Diffusion weighted imaging* (diffusionsgewichtete Bildgebung) |
| DWI-Parameter | Sammelbezeichnung der MRT-Messparameter in der Diffusionsbildgebung, bspw. *apparent diffusion coefficent (ADC)* |
| DPD-Mutation | Dihydropyrimidin-Dehydrogenase Exon 14 Skipping Mutation |
| eCRF | *electronic Case Report Forms* (elektronische Patientendatenbank) |
| EK | Einschlusskriterien |
| EMVI | *extramural vascular invasion* (extramurale Gefäßinfiltration) |
| EORTC | *European Organisation for Research and Treatment of Cancer* |
| FOLFOX4 | Chemotherapie aus 5 Fluorouracil (5-FU), Oxaliplatin, Leucovorin und Folinsäure |
| GFR | Glomeruläre Filtrationsrate |
| Gy | *Gray* (Maßeinheit der durch ionisierende Strahlung transferierten Energiedosis) |
| Hüft-TEP | Hüft-Total-Endoprotese |
| KPS | Karnofsky Performance Status |
| LK+ | Suspekte / befallene Lymphknoten |
| MRT | Magnetresonanztomografie |
| MSP+ | Infiltration der Mesorektalen Faszie (*meso-rectal plane*) |
| PE | Probenentnahme |
| PET-CT | Positronen-Emissionen-Tomografie-CT |
| PFS | Progression free survival |
| PNP | Polyneuropathie |
| Port-System | Subkutan implantiertes, zentralvenöses Zugangssystem |
| RChT | Radiochemotherapie |
| ROC | *Receiver operating characteristic* (Operationscharakteristik) |
| ROI | *region of interest* (Bereich von Interesse / relevante Messregion) |
| TME | Totale mesorektale Exzision (Form der Rektumexstirpation) |
| TILs | *tumor infiltrating lymphocytes* (tumorinfiltrierende Lymphozyten) |
| TNM | Staging des Tumors entsprechend der *UICC* |
| TNT | Totale neoadjuvante Therapie (9 Zyklen FOLFOX) |
| TRG | Tumor Regression Grading |
| UICC | Internationale Vereinigung gegen Krebs (*franz.* Union internationale contre le cancer) |
| V0, …, V11 | Visite 0, …, Visite 22 |
| Z. n. | Zustand nach |

# 2. Fragestellung bzw. Hintergrund

## 2.1 Therapie des Rektumkarzinoms

Das Rektumkarzinom stellt eine häufige Erkrankung in der deutschen Bevölkerung dar. Jede dritte Tumorerkrankung des Mannes und sogar jede zweite bei der Frau gehen auf Darmkrebserkrankungen zurück, wovon die Hälfte der Fälle im Rektum lokalisiert ist. Die Inzidenz steigt mit zunehmendem Alter. Es wurden 2018 über 60.000 Neuerkrankungen diagnostiziert; aufgrund des demographischen Wandels ist jedoch in den nächsten Jahren von einer zunehmenden Inzidenz auszugehen [2].

### 2.1.1 Etablierte Therapiekonzepte und deren Weiterentwicklung

Die Lebensqualität und Prognose der Patienten ist durch die moderne Therapieverfahren deutlich verbessert worden [3]. Die standardmäßige Therapie in den lokal fortgeschrittenen UICC4-Stadien II / III besteht aus einer neoadjuvanten Radiochemotherapie (RChT), gefolgt von einer Operation [3]. Dieses Vorgehen erhöht insbesondere die lokale Kontrolle des Tumors bei lokal fortgeschrittenem Tumorbefund [4]. Diese Behandlung wird aktuell um eine zusätzliche päoperative Systemtherapie ergänzt, wobei sich die simultane Radiochemotherapie gefolgt von einer konsolidierenden Chemotherapie gegenüber der Therapiesequenz einer Induktionschemotherapie gefolgt von simultaner Radiochemotherapie jeweils vor Tumorresektion als überlegen erwiesen hat. Die Radiochemotherapie gefolgt von einer konsolidierenden Chemotherapie führt häufiger (in über 20 % der Patienten) bei vergleichbarer chirurgischer Morbidität zu einer kompletten Remission („complete remission“ / cR) [5-7]. Dieses multimodale Konzept ist als „total neoadjuvante Therapie“ (TNT) beschrieben. Es zeigten sich jedoch auch Limitationen hinsichtlich der Compliance aufgrund therapiebedingter Nebenwirkungen der Chemotherapie oder des Risikos eines onkologisch unvorteilhaften Verlaufes bei frühem Progress von „non-respondern“ [5]. Das Konzept der TNT ist damit nicht für alle Patientengruppen gleichermaßen geeignet.

International wird die TNT empfohlen, es fehlt jedoch an Evidenz-basierten Empfehlungen hinsichtlich der Therapieadaptation an das patientenindividuelle Tumoransprechen. Eine Therapiedeeskalation mit Verzicht auf die bisher übliche Resektion könnte bei makroskopisch komplettem Tumoransprechen onkologisch sicher sein [8]. Statistisch ließe sich entsprechend der publizierten Daten bei wenigstens einem von fünf Patienten eine Operation zunächst vermeiden. Erste Untersuchungen zeigen, dass dieses *„watch and wait“*-Konzept, d.h. eine Resektion erst bei Nachweis eines (Lokal-) Rezidivs, hinsichtlich des Krankheitsverlaufes onkologisch zu vertreten ist [9]. Der Verzicht auf eine direkte Operation verhindert gerade in frühen Stadien (I-II nach UICC4) eine Übertherapie und kann zu einer verbesserten Lebensqualität führen [8].

Ob eine Adaptation der Radiochemotherapie als Therapiedeeskalation oder –eskalation (z.B. Anpassung des Zielgebiets, Dosiserhöhung in makroskopischem Tumorrest) an das Tumoransprechen sinnvoll ist, wurde bisher nicht untersucht. Es gibt lediglich Arbeiten, in denen eine solche Dosiseskalation im Tumor (sog. „Boost“) während der neoadjuvanten RChT mit vielversprechenden Ergebnissen hinsichtlich höherer Raten an cR untersucht wurden [10]. Aktuell ist diese Dosiseskalation im Rahmen der neoadjuvanten Therapie hierzulande nicht etabliert. Mit steigender Dosis sind vermehrte therapiebedingte Nebenwirkungen in den angrenzenden Risikoorganen nicht auszuschließen. Zudem bietet im neoadjuvanten Konzept ein „Boost“ hinsichtlich des Outcomes keinen Benefit, da das Rektum anschließend entfernt wird [10]. Eine solche Dosiseskalation ist allerdings für Patienten, die bei gutem Ansprechen auf die RChT potenzielle Kandidaten eines „watch and wait“ Konzeptes sind und von besseren Aussichten auf eine cR durch den Boost profitieren, von großem Interesse.

Entscheidend für die Weiterentwicklung der bisherigen multimodalen Therapie zu einem individuellen Konzept durch Adaptation der Therapie an den Therapieverlauf ist eine verlässliche Prädiktion des

[4] Union internationale contre le cancer

Ansprechens zu definierten Zeitpunkten während der Therapie. Aktuell stützt sich diese Entscheidung lediglich auf die makroskopische Beurteilung in der klinischen Untersuchung bzw. Rektoskopie sowie MRT des Beckens. Mikroskopische Restbefunde lassen sich damit nicht zuverlässig nachweisen, sind jedoch ein potenzielles Risiko für einen onkologisch nachteiligen Verlauf (bspw. Fernmetastasierung aufgrund des Verzichts auf die Rektumexstirpation) [8]. Die standardmäßig verwendeten MR-Sequenzen bieten letztlich insbesondere volumetrische Informationen und sind dadurch in ihrer Aussagekraft eingeschränkt: Unter der Therapie kommt es erst verzögert zur Volumenreduktion, zudem sind kaum Aussagen zur Zellviabilität möglich [11, 12].

Es ist beschrieben, dass sich in den Tumoren molekulare Unterschiede finden lassen, die mit unterschiedlichem Therapieansprechen einhergehen [13]. Jedoch ist bisher daraus keine Ableitung einer adaptiven Therapieempfehlung möglich [14]. Mittels Tumor-infiltrierender Lymphozyten (TILs) gelingt ein Nachweis der antitumoralen Aktivität des Immunsystems [15, 16]. Insbesondere CD8-positive Lymphozyten scheinen einen unabhängigen Marker zur Immunantwort darzustellen, der sowohl mit der Gesamtprognose des Patienten zum Gesamtüberleben, als auch der cR, korreliert ist [16-18]. Ähnlich dazu lässt sich aus der Analyse im Blut zirkulierender Tumorzellen (CTCs) potentiell die systemische Tumorkontrolle und das Metastasierungsrisiko ableiten [19]. TILs und CTCs könnten damit auch potentiell Therapieentscheidungen unterstützen.

### 2.1.2 Rolle der MR-Bildgebung in der neoadjuvanten Therapie

Mit den geplanten MR-Sequenzen soll durch Abbildung von Diffusionsrestriktionen und hypoxischen Arealen das sog. „Microenvironment“ des Tumors unter der Therapie dargestellt werden.

Die MR-Diffusionsbildgebung (DWI) ist eine nicht invasive Methode, die zur Abschätzung des Tumoransprechens geeignet scheint. Sie erlaubt indirekte Rückschlüsse auf Stoffwechselprozesse und die Integrität von Zellverbänden im Körper [11]. Durch die DWI wird die konventionelle MR-Bildgebung um Informationen zu pathologischen Veränderungen des Stoffwechsels bzw. der Zellviabilität erweitert. Damit ist auch im Therapieverlauf eine bessere Diskriminierung bei suspekten Befunden zwischen tumorbedingten und therapeutischen Veränderungen möglich [20].

In Studien ohne zusätzliche konsolidierende Chemotherapie (keine „TNT“) sind vielversprechende erste Ergebnisse zur Darstellung des Ansprechens mittels DWI publiziert worden [11, 12, 21]. Bisher wurden jedoch noch kaum Untersuchungen zur nicht-invasiven Abschätzung des Tumoransprechens im Rahmen der TNT veröffentlicht und es gibt keine validen Grenzwerte, um beispielsweise anhand des „*apparent diffusion coefficients* (ADC)“ eine objektive Diskriminierung zwischen gutem und schlechtem Therapieansprechen zu ermöglichen.

Ein entscheidender Mechanismus von Strahlenresistenz ist die Hypoxie in Tumorzellen [22, 23]. Diese im Behandlungsverlauf mittels Hypoxie-sensitiver T2*-Sequenzen abzubilden soll das Ziel zur Identifikation eines weiteren, MR-gestützten Surrogatmarkers sein.

Eine solche MR-gestützte Identifikation von gutem Tumoransprechen ist insbesondere von Interesse, um die Patientengruppe, die von einer TNT maximal profitieren kann, besser einzugrenzen. Dadurch würde eine entsprechende Adaptation des Therapieschemas an die individuelle Tumorbiologie bzw. das individuelle Tumoransprechen für jeden Patienten ermöglicht.

## 2.2 Fragestellung und Begründung des Vorhabens

Die Etablierung der DWI im Rahmen der neoadjuvanten RChT ist insbesondere von Interesse, um die Patientengruppe, die von einer TNT maximal profitieren kann, besser einzugrenzen. Das Ziel sollte perspektivisch sein, bei Patienten mit einem sehr guten Ansprechen nach initialer RChT durch eine anschließende individuelle Adaptation des Therapiekonzeptes, die Chance auf eine cR zu erhöhen. An dieser Stelle soll die Pilotstudie ansetzen. Es sollen erste Untersuchung durchgeführt werden, um das prognostische Potenzial wiederholter DWI im Therapieverlauf zur nicht-invasiven Differenzierung von Patienten mit gutem Therapieansprechen von solchen mit schlechtem Ansprechen im

Therapieverlauf zu prüfen und damit die Grundlage für die Entwicklung adaptiver Therapiekonzepte in Folgeprojekten zu legen.

## 2.3 Begründung der Behandlungs- und Untersuchungsverfahren

Die MRT-Signalveränderungen können als Surrogatmarker Stoffwechselvorgänge abbilden und ermöglichen damit direkte Rückschlüsse auf die mikroskopische Beschaffenheit des Gewebes hinsichtlich Tumorresten. Damit bieten sie im Vergleich zu den aktuell verwendeten MRT-Sequenzen ein Informationsplus, insbesondere hinsichtlich der Zellviabilität. Dies ist in der betrachteten Fragestellung eine essentielle Voraussetzung, da mikroskopische Tumoranteile ansonsten erst mit der Operation histologisch nachzuweisen sind. Eine PET-CT kann ähnliche Informationen liefern, jedoch ist der apparative Aufwand größer und es kommt zur zusätzlichen Strahlenbelastung des gesamten Körpers aufgrund des verwendeten Tracers und der CT-Schnittbildgebung mit Röntgenstrahlung. Dahingegen kommt die MRT ohne ionisierende Strahlung aus.

Mit zusätzlichen histologischen und serologischen Markern (TILs, CTC) lässt sich die Dynamik des Tumors im Verlauf der neoadjuvanten Therapie darstellen. Diese Erkenntnisse ermöglichen Rückschlüsse auf die Prognose des Patienten und können perspektivisch bei der Therapieentscheidung (bspw. Intensivierung oder Deeskalation) von Relevanz werden.

# 3. Studienziele und Endpunkte

Übergeordnetes Ziel dieser prospektiven Pilotstudie ist die Evaluation der prädiktiven Wertigkeit von multimodaler Diagnostik, wie MR-Aufnahmen, zirkulierender Tumorzellen (CTC), Tumor-infiltrierender Lympozyten (TILs), im präoperativen Verlauf hinsichtlich des Therapieansprechens und ihrer Assoziation mit dem histologischen Korrelat des Resttumors. Ferner wird die Dauer und der Umfang der neoadjuvanten Therapie hinsichtlich des Tumoransprechens und der Therapieverträglichkeit untersucht.

## 3.1 Primäres Studienziel und primärer Endpunkt

Das primäre Studienziel ist die Untersuchung der prädiktiven Wertigkeit der longitudinalen *MR-Bildgebung* für das histologische Therapieansprechen auf eine neoadjuvante 5-Fluorouracil (5-FU)-basierte Radiochemotherapie (RChT) +/- konsolidierende Chemotherapie (ChT) mit FOLFOX4 als total neoadjuvante Therapie (TNT).

Somit ist der primäre Endpunkt das Ansprechen auf die neoadjuvante Therapie. Das Ansprechen wird mittels der histologischen Untersuchung des Resektates oder von seriellen Biopsien (ypTNM) und des TRG nach TU München [24, 25] ermittelt. Ein Ansprechen ist mittels TRG-Score binär definiert: Ansprechen: Score von 1a/b oder 2; Nichtansprechen: Score von 3 [26].

## 3.2 Sekundäre Studienziele und sekundäre Endpunkte

Sekundäre Ziele sind die Volumetrie des Tumors im Verlauf und Identifikation des frühestmöglichen Nachweises eines Tumoransprechens nach neoadjuvanter Therapie anhand von MR DWI- und Hypoxie-Parametern, Untersuchung von Veränderungen des Tumormicroenvironments (TILs) und Identifikation von strahlentherapiebedingten Veränderungen im tumorfreien Rektum. Zudem sollen Follow-up-Parameter und Lebensqualität untersucht werden. Sekundäre Zielgrößen sind daher:

- Vorhandensein und Volumen eines Resttumors nach neoadjuvanter RChT +/- anschließender konsolidierender ChT („TNT“-Konzept)
- Differenziertere Definition des Therapieansprechens (ordinal) entsprechend ypTNM und TRG sowie Komplettremission (cR), definiert als ypT0N0M0 bzw. TRG 1a/b
- Longitudinale multiparametrische MR-Signalveränderungen
- MR-Signalveränderungen in einer tumorfreien *region of interest* des Rektums als Referenzstruktur
- Veränderungen des Phänotyps der Tumorzellen nach neoadjuvanter RChT, insbesondere exprimierter Oberflächenproteine
- Analyse von TILs und deren Phänotyp (insb. exprimierte Oberflächenproteine) in der prätherapeutischen Probe sowie im Resektat der Operation
- Longitudinale Veränderungen der Zahl und des Phänotyps (insb. exprimierte Oberflächenproteine) der CTCs und in der zirkulierenden Tumor-DNA (ctDNA)
- Therapiebedingte Nebenwirkungen nach EORTC CTCAE
- Lebensqualität nach EORTC QLQ-C30 bzw. QLQ-CR29 im Therapieverlauf und Follow up
- *Progression free* survival (PFS) sowie Therapieversagen *(„treatment failure“)* i.S. von Lokalrezidiv oder Fernmetastasen
- 5 Jahres Überlebensrate *(„overall survival“,* OS)

# 4. Studiendesign und -beschreibung

## 4.1 Art der Studie

Es handelt sich um eine offene, prospektive, nicht randomisierte, monozentrische Pilotstudie, in der die Eignung multimodaler Diagnostik zur Vorhersage des Tumoransprechens geprüft wird. Diese Studie unterliegt den Kriterien einer Studie nach §15 der Berufsordnung für Ärzte.

## 4.2 Art der Therapiezuordnung

Alle eingeschlossenen Patienten erhalten eine leitliniengerechte simultane neoadjuvante Radiochemotherapie. Nach deren Abschluss wird die Chemotherapie mit FOLFOX4 entsprechend der einer totalen neoadjuvanten Therapiefortgeführt, wenn klinisch indiziert (vgl. Punkt 0) und im Rahmen des multimodalen Konzeptes verträglich.
Das Blutbild muss zu Beginn des ersten Zyklus der TNT die in Abschnitt 6.5 definierten Kriterien zur Durchführung der Chemotherapie erfüllen (Neutrophile ≥3 x $10^9$/l, Thrombozyten ≥100 x $10^9$/l, Hämoglobin ≥6 mmol/l). Auf Patientenwunsch kann, trotz klinischer Indikation für die Durchführung der TNT, auf die zusätzliche konsolidierende Chemotherapie verzichtet werden.

Alle Patienten, die eine vollständige TNT mit 9 Zyklen FOLFOX4 erhalten, werden nach 34 Wochen operiert oder bekommen bei gutem Therapieansprechen (klinische Komplettremission im Restaging) optional ein organerhaltendes Konzept angeboten. Bei den Patienten, wo aufgrund der therapiebedingter Nebenwirkungen oder aus anderen Gründen keine Fortführung der TNT möglich ist, erfolgt die Operation nach 16 Wochen. Bei diesen Patienten ist kein organerhaltendes Konzept möglich (siehe Abschnitt 6.4).

## 4.3 Indikationen zur TNT

Nach Abschluss der kombinierten RChT wird folgenden Patienten die Fortführung einer konsolidierenden Chemotherapie analog den Empfehlungen der konsentierten Stellungnahme der ARO, AIO und ACO von 2020 [27] angeboten:

- Adenokarzinom entsprechend der Einschlusskriterien, darüber hinaus gehend jedoch:
  - mindestens uT3, cN0 der cN+, cM0 (UICC Stadium II bis III)
  - jedes cT3 bei tiefem Sitz
  - bei T3 mit Tumor im mittleren Rektumdrittel bei Vorliegen mindestens einer der zusätzlichen Faktoren: positive Lymphknoten (N+); Infiltration des mesorektalen Fettgewebes >5mm (cT3c/d) bzw. der mesorektalen Faszie (MSP+);extramurale Gefäßinvasion (EMVI+)
  - jedes T4
- ausreichend gutes Blutbild entsprechend der in Abschnitt 6.5 definierten Kriterien

Für das Vorgehen bei Kontraindikationen zur TNT bzw. dem Abbruch der TNT siehe Kapitel 6.5.

## 4.4 Zahl und Art der Vergleichsgruppen

Patienten mit einer vollständigen konsolidierenden Chemotherapie (TNT, 9 Zyklen FOLFOX4) bilden die Analysegruppe I. Patienten, bei denen die TNT nicht (komplett) durchgeführt werden kann, bilden die Analysegruppe II.

## 4.5 Patientenrekrutierung / Umfang der Studie

Die Studie soll monozentrisch in der Klinik für Strahlentherapie und Radioonkologie am Universitätsklinikum Jena durchgeführt werden. Es werden Patienten von drei extern zuweisenden Krankenhäusern[5] sowie hausintern[6] rekrutiert. Die Patienten werden im Rahmen des

---

[5] Klinik für Allgemein-, Viszeral- und Thoraxchirurgie / Thüringenkliniken Saalfeld; Klinik für Allgemein-, Viszeral und Gefäßchirurgie /Sophien- und Hufelandklinikum Weimar; Klinik für Allgemein- und Viszeralchirurgie, SRH Klinikum Naumburg

Aufklärungsgespräches zur Bestrahlungsplanung auf die Studie hingewiesen. Im Fall von Interesse wird ein Aufklärungstermin mit einem der Prüfärzte vereinbart.

Es wird von ca. 25 Patienten jährlich mit Rektumkarzinom in den Stadien II-III, die eine Indikation zur neoadjuvanten Radiochemotherapie mit 5-FU / Oxaliplatin haben, ausgegangen. Somit werden in einem Rekrutierungszeitraum von zwei Jahren 50 Patienten mit Rektumkarzinom erwartet, von denen voraussichtlich 15 die vollständige konsolidierende Chemotherapie (9 Zyklen FOLFOX4) durchlaufen werden. Für weitere Details sei hier auf den Abschnitt 8.3 zur Fallzahlplanung verwiesen.

## 4.6 Zeitplan

Die Durchführung der Pilotstudie erfolgt im Rahmen der *„clinician scientist“*-Förderung des IZKF. Die patientenbezogene Dauer bis zum Erreichen des primären Endpunktes bei vollständiger konsolidierender Chemotherapie mit 9 Zyklen FOLFOX 4 beträgt ca. 9 Monate. Bei einer Rekrutierungsdauer von ca. 2 Jahren ist die Auswertung des primären Endpunktes 3 Jahre nach dem Studieneinschluss des ersten Patienten geplant. Es folgt die Erhebung der weiteren sekundären Endpunkte im Rahmen der 5-jährigen Nachbeobachtung. Die Gesamtstudiendauer (*first patient, first visit – last patient, last visit*) inklusive der Auswertung soll daher 8 Jahre betragen.

Daraus ergibt sich folgender vorläufiger Zeitplan für die prozessbezogene Studiendauer:

| | |
|---|---|
| Rekrutierungsdauer: | ca. 24 Monate |
| Einschluss erster Patient: | Q4/2022 |
| Einschluss letzter Patient: | Q4/2024 |
| Letztes Follow-up: | Q3/2030 |
| Auswertung primärer Endpunkt: | Q3/2025 |
| Auswertung: | Q4/2030 |
| Abschlussbericht: | ca. 6 Monate nach letztem Follow-up |
| Gesamtdauer: | 8 Jahre |

[6] Klinik für Allgemein-, Viszeral- und Gefäßchirurgie (AVGC), UKJ

# 5. Studienpopulation

Es gelten **folgende Einschlusskriterien** (und keine zusätzlichen Ausschlusskriterien):

- histologisch bestätigtes Adenokarzinom entsprechend der Einschlusskriterien des unteren (0-6 cm ab ano) oder mittleren Rektumdrittels (6-12 cm ab ano) [7]:
    - mindestens uT3, cN0 der cN+, cM0 (UICC Stadium II bis III)
    - jedes cT3 bei tiefem Sitz
    - bei T3 mit Tumor im mittleren Rektumdrittel bei Vorliegen mindestens einer der zusätzlichen Faktoren: befallene perirektale Lymphknoten (N+); Infiltration des mesorektalen Fettgewebes >5mm (cT3c/d) bzw. der mesorektalen Faszie (MSP+); extramurale Gefäßinvasion (EMVI+)
    - jedes T4
    - bei Sitz im oberen Rektumdrittel nur bei besonderen Risikofaktoren für eine zu erwartende R1-Situation oder ausgeprägtem Lymphknotenbefall: T4, MSP+ oder N2 (entsprechend der Empfehlung der interdisziplinären Tumorkonferenz)
- Indikation zur neoadjuvanten Radiochemotherapie sowie ggf. anschließender konsolidierender Chemotherapie als total neoadjuvante Therapie (TNT) vor operativer Resektion
- Alter ≥ 18 Jahre
- Karnofsky Performance Status (KPS) 70-100%
- Keine weitere aktive Tumorerkrankung.
    - ausgenommen nicht-invasive Basaliome oder nicht-invasive Plattenepithelkarzinome der Haut, therapierte Prostata-Karzinome bei PSA < 0,2 ng/ml
- Keine vorhergehende Strahlentherapie des Beckens
- Keine vorangegangene Chemotherapie
- Keine schwere Begleiterkrankung:
    - Keine schwere obstruktive oder restriktive Lungenerkrankung
    - Keine schwere allergische Erkrankung, die eine kontinuierliche Medikation erfordert
    - Keine schwere Herzerkrankung (kongestive Herzinsuffizienz, Angina pectoris, neue koronare Herzkrankheit, unkontrollierte Herzrhythmusstörungen oder Leitungsstörungen)
    - Keine schwere Magen-Darm-Erkrankung, aktive peptische Ulkuskrankheit
    - Keine schwere Beeinträchtigung der Leberfunktion (Bilirubin, Transaminasen, alkalische Phosphatase >2,5-Fache der oberen Grenze des Normalbereichs)
    - Keine schwere Beeinträchtigung der Nierenfunktion mit einer glomerulären Filtrationsrate (GFR) <30 ml/min,
    - Keine unkontrollierte endokrine Erkrankung
    - Keine vorbestehende schwere psychische oder schwere organische Hirnerkrankung (z. B. vorbestehende Epilepsie)
- Keine Kontraindikation gegen eine Therapie mit Oxaliplatin oder 5-FU. Folgende hämatologische Werten dürfen nicht unterschritten werden:
    - Neutrophile ≥3 x $10^9$/l
    - Thrombozyten ≥100 x $10^9$/l
    - Hämoglobin ≥6 mmol/l
- vollständiges Staging vor Radiochemotherapie, bestehend aus DRU, Rektoskopie/Koloskopie mit ERUS, MRT Becken (nativ), CT Thorax / Abdomen / Becken mit Kontrastmittel (alternativ, sofern indiziert PET-CT)
- Ausschluss einer homozygoten DPD-Exon-Skipping-Mutation [1]
- Keine psychologischen, familiären, soziologischen oder geografischen Umstände, die die Einhaltung des Studienprotokolls und des Zeitplans für die Nachbeobachtung behindern könnten; diese Umstände sollten mit dem Patienten vor der Registrierung für die Studie besprochen werden.
- Frauen dürfen nicht schwanger sein oder stillen. Frauen und Männer im gebärfähigen Alter, die in die Studie aufgenommen werden, werden darüber aufgeklärt, dass die Behandlung teratogen sein kann. Patienten und Patientinnen müssen zustimmen, während der Radiochemotherapie und Chemotherapie sichere Maßnahmen zur Verhinderung einer Empfängnis zu ergreifen.

[7] gemessen in der starren Rektoskopie

- Vor der Patientenregistrierung muss eine schriftliche Einwilligung des Patienten/der Patientin nach Aufklärung gemäß ICH/GCP erteilt sein.
- Keine parallele Teilnahme an einer anderen interventionellen klinischen Prüfung
- Keine Kontraindikation für MRT:
  - Keine Herzschrittmacherimplantation
  - Keine Hüft-total-Endoprothese (Hüft-TEP)
  - Keine Metallimplantate von >15 cm Länge
  - Kein nicht MR-gängiges Port-System
  - Keine ausgeprägte Adipositas (BMI >30 kg/m²)

# 6. Studienablauf

## 6.1 Screening und Patientenidentifikationsliste

Patienten mit Rektumkarzinom, welche in der Klinik für Strahlentherapie und Radioonkologie vorstellig werden, werden täglich durch einen Prüfarzt hinsichtlich der oben genannten Kriterien für den potentiellen Studieneinschluss untersucht. Patienten, die die Einschlusskriterien erfüllen, werden im Screening-Log gelistet. Patienten, die in die Studie eingeschlossen wurden, werden in die Patientenidentifikationsliste aufgenommen.

## 6.2 Aufklärung und Einwilligung

Die Studienteilnahme ist freiwillig. Die Aufklärung der Patienten über Wesen, Bedeutung, Ziele, mögliche Risiken, erwartete Vorteile, Tragweite und sonstige Aspekte der klinischen Prüfung erfolgt mit der vorgegebenen Patienteninformation und Einwilligungserklärung sowie durch ein Gespräch mit einem Arzt des Studienteams. Die Einwilligung erfolgt schriftlich auf dem vorgegebenen Formular. Der Prüfer überzeugt sich davon, dass die Aufklärung vom Patienten verstanden wurde. Nach der Aufklärung erhält jeder Patient ausreichend Zeit und Gelegenheit, offene Fragen zu klären und über seine Teilnahme zu entscheiden.

Jeder Patient unterzeichnet und datiert seine Einwilligung in die Teilnahme an der Studie eigenhändig schriftlich auf der Einwilligungserklärung. Die Einwilligung des Patienten muss sich ausdrücklich auch auf die Erhebung und Verarbeitung von personenbezogenen Daten beziehen. Deshalb werden die Patienten explizit über Zweck und Umfang der Erhebung und die Verwendung dieser Daten, insbesondere von Gesundheitsdaten, informiert.

Es werden nur einwilligungsfähige Patienten eingeschlossen. Ist ein einwilligungsfähiger Patient nicht in der Lage, eigenhändig die Einwilligung zu unterzeichnen, muss ein Zeuge, welcher nicht Mitglied des Studienteams sein darf, während des Aufklärungsprozesses anwesend sein. Dieser Zeuge bestätigt die mündliche Aufklärung und Einwilligung des Patienten durch Datum und Unterschrift.

Ein Exemplar der unterschriebenen Patienteninformation und Einwilligungserklärung (Kopie oder zweites Original) wird dem Patienten ausgehändigt, das andere verbleibt im Prüfzentrum und wird im Prüfarztordner aufbewahrt.

Der Patient kann jederzeit und ohne Angabe von Gründen die Einwilligung zurückziehen und die Behandlung abbrechen bzw. die Studie abbrechen. Der Patient wird in solchen Fällen gebeten, den Abbruchgrund (für die Behandlung oder die Teilnahme) zu nennen, jedoch darauf hingewiesen, dass er dies nicht tun muss. Der Zeitpunkt der Rücknahme der Einwilligung zur Studie ist zu dokumentieren (vgl. auch Abschnitt 6.7).

## 6.3 Studienverlauf

Nach Prüfung der Einschlusskriterien erfolgt die Aufklärung und schriftliche Einwilligung im Rahmen einer Konsultation an der Klinik für Strahlentherapie und Radioonkologie.

Vor Beginn der RChT erfolgt die erste studienspezifische MRT sowie eine Blutentnahme zur Analyse der CTCs und des Tumormarkers CEA, anschließend die leitliniengerechte Radiochemotherapie. Die studienspezifischen Untersuchungen sind im Visitenplan im Abschnitt 1.6 aufgelistet. Nach Abschluss der Radiochemotherapie wird die zweite MRT durchgeführt und in einer erneuten Blutentnahme CTCs sowie das CEA bestimmt (V5 / Woche 8).

Anschließend wird bei entsprechender Indikation und Einverständnis des Patienten die Chemotherapie nach FOLFOX4 Schema fortgeführt. Hierfür ist ein ausreichend gutes Blutbild sowie eine ausreichende Nierenfunktion, analog der Einschlusskriterien zu Beginn der Studie, notwendig (vgl. Indikation zur TNT, Abschnitt 0). Sofern diese Kriterien nicht erfüllt sind oder der Patient die weitere Chemotherapie ablehnt, wird bis zur Operation nach 8 Wochen nachbeobachtet und eine dritte MRT präoperativ (V7, Woche 14) durchgeführt.

Die Chemotherapie mit FOLFOX4 erfolgt im 2-wöchigen Rhythmus. Im Rahmen der konsolidierenden Chemotherapie sind drei MRTs vorgesehen.

Bei Dosisreduktion, zeitlich protrahierter Gabe oder Ausfall eines Chemotherapiezyklus aufgrund therapieassoziierter Nebenwirkungen (vgl. Abschnitt 6.5) wird der oben definierte Visitenplan beibehalten. Wird die neoadjuvante Chemotherapie vor Ende des 9. Zyklus abgebrochen bzw. die planmäßige Operation vorgezogen, so wird im Rahmen des prä-operativen Stagings eine MRT nach Studienprotokoll angefertigt und weitere MRTs entfallen (analog Analysegruppe II; siehe Abschnitt 6.7). Die TNT gilt als vollständig appliziert, wenn über den Zeitraum von 18 Wochen mindestens 8 von 9 Zyklen durchgeführt wurden.

Bei allen Patienten ist routinemäßig ein prä-operatives Staging vorgesehen, im Rahmen dessen die letzte studienspezifische MRT sowie eine Blutentnahme (CTCs, CEA) erfolgen. Anschließend werden die Patienten in der zuweisenden chirurgischen Klinik zur Resektion des Rektums vorstellig. Optional ist nach Abschluss der TNT bei klinischer Komplettremission (cCR) analog der ARO/AIO/ACO Stellungnahme zum Organerhalt [28] ein abwartendes Vorgehen unter engmaschiger Nachsorge möglich. In diesem Fall müssen bei der Endoskopie serielle Biopsien (mind. 5 Stück [29]) erfolgen. Dies gilt nicht für Patienten, die keine TNT erhalten. Die Präparate der Resektion oder ggf. der seriellen Biopsien werden in den kooperierenden pathologischen Instituten vorbereitet und befundet. Sie werden anschließend zur Referenzbefundung hinsichtlich der Tumorregression und studienspezifischen Untersuchungen (insbs. TILs) an das Institut für Pathologie am Universitätsklinikum Jena versandt.

Im Anschluss an die Resektion erfolgt die Nachbeobachtung der Patienten entsprechend der gesetzlichen Nachsorgefrist von 5 Jahren. Hierfür sind regelmäßige klinische Kontrollen sowie Blutentnahmen zur Bestimmung der CTCs entsprechend des Visitenplanes vorgesehen.

## 6.4 Studienbedingte Maßnahmen

Folgende studienbedingte Maßnahmen erfolgen zu den aufgeführten Zeitpunkten (siehe Tabelle 1: Visitenplan):

**Probenentnahme mit histologischer Aufarbeitung (initiales Staging)**
Zeitpunkt: Screening (4 Wochen vor Studienbeginn)
Entnahme und Aufarbeitung: Die bei der histologischen Sicherung des Tumors gewonnenen Tumorschnitte werden in der anfertigenden Pathologie hinsichtlich des Stagings routinemäßig ausgewertet. Es erfolgt eine Zweitbefundung am Institut für Pathologie des UKJs hinsichtlich der TILs in Tumorumgebung und Oberflächenproteinen der Tumorzellen (bspw. PD-L1).

**Forschungs-MRT** inkl. DWI und Hypoxie-sensitiven Sequenzen
Im Rahmen des Protokolls wird aus dieser zusätzlichen Bildgebung keine therapeutische Konsequenz für individuelle Patienten abgeleitet.
Zeitpunkte: Woche 2-3 (V1), Woche 5 (V3), Woche 8 (V5), Woche 14 (V8), Woche 20 (V11, nur für Patienten in Analysegruppe I), Woche 32 (V15, nur für Patienten in Analysegruppe I)
Messung: Es wird ein 3 T MR-Scanner (Siemens „Vida“, body array, spine Spule) verwendet und diffusions-gewichtete T2-Sequenzen ohne Kontrastmittel angefertigt. Die Untersuchungen werden im Universitätsklinikum Jena (Standort Lobeda) durchgeführt.
Die Messungen erfolgen in Anwesenheit eines erfahrenen medizinisch-technischen Mitarbeiters des IDIR, der mit der Bedienung des MRT-Gerätes vertraut ist, und von einem Mitarbeiter der AG „Medizinische Physik“ zur Durchführung der studienspezifischen Sequenzen geschult wurde. Die Durchführung der MRTs erfolgt zudem unter Aufsicht eines Arztes. Vor der Messung werden nicht-MR-kompatible i.v. Infusionssysteme entfernt (z.B. 5-FU Dauerinfusionssysteme) und im Anschluss wieder in Betrieb genommen. Die Bildgebung erfolgt mit leerem Rektum. Zur Reduktion von intestinalen Bewegungsartefakten erfolgt eine Prämedikation mit Buscopan p.o / i.v. nach Ausschluss von Kontraindikationen. Bei Vorliegen einer der folgenden Nebendiagnosen wird auf die Applikation verzichtet:

- Engwinkelglaukom
- Tachykardie / Tachyarryhthmia absoluta
- Gastrointestinale Obstruktionen / Megakolon
- Obstruktionen der ableitenden Harnwege
- Myasthenia gravis

Auswertung: Die Auswertung beinhaltet die Messung des ADCs und weiterer mikrostrukturbeschreibende Diffusions- und Hypoxieparameter, die im Folgenden zusammengefasst als „MR-Parameter“ benannt sind. Diese werden im Primärtumor des Rektums sowie exemplarisch in suspekten mesorektalen Lymphknoten bestimmt.
Zusätzlich wird in jedem MRT eine 0,5 cm lange Referenzstruktur von 0,75 cm bis 1,25 cm kranial des Tumoroberrandes aber im Zielgebiet der Strahlentherapie definiert, in der ebenfalls DWI-Parameter aufgezeichnet werden.

**Lebensqualitätsfragebogen und CTCAE-Bogen**
Zeitpunkte: Woche 2-3 (V1), Woche 8 (V5), Woche 24 (V13, nur für Patienten in Analysegruppe II), Woche 42 (V16)
Erhebung: Die EORTC QLQ-C30 / QLQ-CR29-Fragebögen werden den Patienten ausgehändigt und von diesen selbstständig ausgefüllt. Die Erhebung und Graduierung der therapiebedingte Nebenwirkungen anhand des organbezogenen CTCAE-Bogen nach EORTC CTCAE v5.0 (vgl. Anlage 11.3) erfolgt in der Klinik für Strahlentherapie und Radioonkologie.

**Blutentnahmen zur Erhebung der Laborwerte**

Bestimmung des CEA und IL-6: bei routinemäßigen Blutentnahmen im Rahmen des Screenings sowie in Woche 8 (V5), sowie in Woche 16 (V9 / präoperativ in Analysegruppe II) und präoperativ in der Analysegruppe I in Woche 34 (V14). CEA anschließend regelmäßig im Rahmen der Nachsorgen.

Bestimmung von CTCs und ctDNA
Hierfür werden vor Therapiebeginn (Screening), nach RChT (V5, Woche 8), präoperativ (Analysegruppe I: V14, Woche 32, Analysegruppe II: V7, Woche 14) sowie im Rahmen der Nachsorge (8 Wochen, 6 Monate, jährlich bis 5 Jahre) bei routinemäßigen Blutentnahmen jeweils ein zusätzliches EDTA-Röhrchen (Vollblut) abgenommen und im kooperierenden Labor der AG Dr. Clement / Klinik für Hämatologie und Internistische Onkologie, Universitätsklinikum Jena ausgewertet. Die Messung erfolgt analog einer aktuellen Studie am Universitätsklinikum Jena (Ethikvotum 2020-1987-Material). Es werden die absolute Zellzahl bestimmt und Oberflächenproteine (wie bspw. PD-L1) analysiert.
Sofern im Rahmen dieser Auswertungen Probenreste anfallen, werden diese entsprechen der Allgemeinen Vertragsbedingungen (AVB) des UKJ §16 aufbewahrt und können für weitere Analysen (bspw. ctDNA) verwendet werden.

**Restaging**

Nach Abschluss der neoadjuvanten Therapie erfolgt zur OP-Vorbereitung ein Re-Staging mit CT Thorax/Abdomen/Becken sowie Rektoskopie, ggf. mit rektalem Ultraschall, in der zuweisenden Klinik.

Organerhaltendes Vorgehen:
Bei klinischer Komplettremission und Patientenwunsch nach vollständiger TNT ist alternativ ein organerhaltendes Vorgehen im Sinne des „watch and wait“-Konzepts möglich. In diesem Fall erfolgen serielle Biopsien (mind. 5 Stück) sowie nach Möglichkeit eine Bürstenzytologie aus dem ehemaligen Tumorbett [29] [30] und engmaschige Kontrollen über die behandelnde chirurgische Klinik. Bei diskordantem Ergebnis der einzelnen Biopsien wird die Biopsie mit der geringsten Tumorregression (dem höchsten ypTNM) zur Definition des Tumoransprechens und zur Festlegung des weiteren Procederes herangezogen. Ein „watch and wait“-Konzept ist ausschließlich bei TRG = 1a/b nach TU München möglich (vgl. Abschnitt 8.1).

**Rektumexstirpation**
Zeitpunkt: Woche 30 (Analysegruppe I) bzw. Woche 16 (Analysegruppe II)
Entnahme und Aufarbeitung:
Nach Abschluss der neoadjuvanten RChT erfolgt die routinemäßige Rektumexstirpation in der zuweisenden Klinik. Das Resektat wird dabei mit mindestens zwei Fadenmarkierungen (bspw. kranial, ventral) versehen, die eine eindeutige anatomische Orientierung ermöglichen. Es wird anschließend in der mit der operierenden Klinik kooperierenden Pathologie aufgearbeitet und ausgewertet.
Entsprechend des Protokolls werden zusätzliche Schnitte in der ehemaligen Tumorregion sowie in der Referenzstruktur 1 cm kranial des Tumors angefertigt und befundet.

**Histologische Aufarbeitung (TRG)**
Die Schnitte / Biopsiepräparate werden baldmöglichst an die Referenzpathologie (Universitätsklinikum Jena) versendet. Dort erfolgt die Auswertung der Regression des Tumors (ypTNM, TRG nach TU München [24, 25]) sowie die Bestimmung der TILs und IELs in Tumornähe. Zudem werden erneut Oberflächenproteine der Tumorzellen analysiert. Die Referenzstruktur wird hinsichtlich histo-morphologischer Veränderungen im Sinne von Ödem, Fibrose- und Entzündungszeichen semiquantitativ ausgewertet.

## 6.5 Laborkontrollen und Dosisreduktion der TNT

Es erfolgen mindestens wöchentliche Laborkontrollen (Blutbild, Nierenfunktion) während der RChT sowie während der anschließenden TNT nach klinikinternem Standard.
Entsprechend dieser Kriterien wird vor jedem Chemotherapie-Zyklus die Therapieverträglichkeit geprüft. Die Chemotherapie wird in der vorgesehenen Dosierung und Zeit appliziert wenn:

keine Nebenwirkungen nach CTCAE °III oder höher UND
Karnofsky-Index ≥70% UND
Laborparameter wie angegeben:
Neutrophilenzahl ≥ 1,5 Gpt/l ODER Leukozytenzahl ≥ 3,0 Gpt/l
UND Thrombozytenzahl ≥ 100 Gpt/l
UND Nierenfunktion mit einer Clearance (GFR) von ≥30 ml/min

Eine individuelle Dosisreduktion auf 50% oder 75% kann darüber hinaus nach Ermessen des behandelnden Arztes (z.B. antizipierte Toxizität bei zuvor schlechter Verträglichkeit) nach Abstimmung mit dem Studienkoordinator erfolgen.

Weiterhin sollte bei Verschlechterung der Laborwerte unter die o.g. Grenzwerte erfolgt zunächst eine dosisreduzierte Applikation der TNT nach folgendem Schema erfolgen:

Dosisreduktion von FOLFOX4
auf 75% der geplanten Dosierung:
Neutrophilenzahl ≥ 1,5 Gpt/l ODER
3 Gpt/l ≥ Leukozytenzahl ≥ 2,5 Gpt/l ODER
100 Gpt/l ≥ Thrombozytenzahl ≥ 75 Gpt/l

auf 50% der geplanten Dosierung:
1,5 Gpt/l ≥ Neutrophilenzahl ≥ 1,0 Gpt/l ODER
2,5 Gpt/l ≥ Leukozytenzahl ≥ 2,0 Gpt/l ODER
75 Gpt/l ≥ Thrombozytenzahl ≥ 50 Gpt/l

Kontraindikation für FOLFOX4
Neutrophilenzahl ≤ 1,0 Gpt/l ODER
Leukozytenzahl ≤ 2,0 Gpt/l ODER
Thrombozytenzahl ≤ 50 Gpt/l ODER

Karnofsky-Index <70%

Falls bei weiterer Verschlechterung diese Parameter nicht erfüllt werden, wird die Chemotherapie zunächst um eine Woche verschoben. Danach erfolgt eine erneute Kontrolle der Therapiefähigkeit. Ist diese erneut nicht gegeben, entfällt dieser Zyklus der Chemotherapie. Der darauffolgende Zyklus der Chemotherapie wird dann bei entsprechend restituierten klinischen und Laborparametern appliziert und die TNT wird fortgeführt. Sollte die Therapiefähigkeit dann nicht erreicht sein, so wird die TNT abgebrochen und der Patient zur Operation vorgestellt.

**Isolierte Dosisreduktion von Oxaliplatin**
Darüber hinaus kann eine Dosisreduktion von Oxaliplatin aufgrund von Polyneuropathie (PNP) notwendig werden:

Keine Polyneuropathie: planmäßige Applikation
Symptome direkt nach der Applikation bis max. 72h fortbestehend: ab dem 6. Konsolidierungszyklus Dosisreduktion auf 50%-75%
Persistierende Symptome für ≤7d oder keine klinische Relevanz (<CTC°II): Dosisreduktion Oxaliplatin auf 50%;
<u>Klinisch relevante</u> PNP (≥CTC°II) persistiert bis zum nächsten Zyklus: keine Applikation von Oxaliplatin in diesem Zyklus

**Dosisreduktion von 5-Fluorouracil**
Bei Vorliegen einer therapierelevanten DPD-Defizienz ist die Dosis der Chemotherapie in sämtlichen Zyklen entsprechend der humangenetischen Stellungnahme anzupassen.
Darüber hinaus kann eine Anpassung der 5-FU Dosierung bei klinischer Symptomatik i.d.R. ab CTCAE °III (wie bspw. Hand-Fuß-Syndrom, gastrointestinale Nebenwirkungen) notwendig sein.

<u>Abbruchkriterien der TNT:</u>
Fällt ein Zyklus der TNT aufgrund der o.g. „Kontraindikationen für FOLFOX4“ aus und liegen in der folgenden Kontrolle nach 2 Wochen erneut o.g. Kontraindikationen vor, so wird die TNT abgebrochen.

## 6.6 Studienende

Das reguläre Studienende für jeden Patienten ist der Abschluss der letzten Follow-Up-Visite V22 mit Ende der 5-Jahres Nachsorgefrist oder das Versterben.

Durch die Rücknahme der Einwilligungserklärung (Widerruf) kann die Studienteilnahme eines Patienten vorzeitig abgebrochen werden. Kann der Kontakt zu dem Patienten nach der Entlassung aus dem Krankenhaus nicht mehr hergestellt werden, ist die Studie für den Patienten ebenfalls vorzeitig beendet („*lost to follow-up*“).

Das reguläre Gesamtstudienende wird nach Auswertung der Daten aller eingeschlossenen Patienten erreicht.

## 6.7 Vorgehen nach dem (vorzeitigen) Ausscheiden

Zieht ein Patient die Einwilligung zur Teilnahme an der Studie zurück (Widerruf), so werden keine weiteren MRTs angefertigt. Sofern der Patient nicht explizit widerspricht, werden die bisher erhobenen Daten, soweit auswertbar, in die Analyse einbezogen. Andernfalls erfolgt eine umgehende Löschung der Daten.

Die standardmäßige Therapie des Rektumkarzinoms bleibt davon unberührt.

Wird die neoadjuvante Chemotherapie vor Ende des 9. Zyklus FOLFOX4 abgebrochen, so wird die Operation vorgezogen. Das Restaging vor OP wird 8 Wochen nach Abbruch der TNT durchgeführt. Der Patient wird analog des Visitenplanes der Analysegruppe I weiterbeobachtet und im Rahmen des präoperativen Stagings eine MRT nach Studienprotokoll durchgeführt. Zur Wahrung des typischen Ablaufes der neoadjuvanten Therapie soll zwischen Ende der Strahlentherapie und Operation ein

Intervall von 8 Wochen liegen. Die letzte MRT erfolgt dann präoperativ (V7). Diese Patienten werden in der Auswertung gesondert betrachtet.

Da bei Abbruch der TNT von keiner ausreichenden Chemotherapiedosis für eine langfristige Tumorkontrolle auszugehen ist, ist ein nicht-operatives Vorgehen nicht indiziert.

# 7. Unerwünschte Ereignisse

Gegenstand dieser Studie ist die Überprüfung des Ansprechens von Patienten mit Rektumkarzinom auf eine Therapie nach Leitlinie mittels MR-Bildgebung.

Weder das MRT-Gerät, noch die Präparate 5-FU Oxaliplatin oder Folinsäure sind im Rahmen dieser Studie Gegenstand der Untersuchung. Die MR-Bildgebung wird seit vielen Jahren zur Diagnostik von vielen onkologischen Erkrankungen eingesetzt. Die Chemotherapie mit 5-FU / Oxaliplatin sowie FOLFOX4 entspricht den Empfehlungen der Fachgesellschaften ARO, AIO und ACO 2020 [27] in diesem Indikationsgebiet. Für alle Produkte liegen seit Jahren umfangreiche Sicherheits- und Verträglichkeitsdaten zum Einsatz vor. Insofern kann für die Studie von einer sicheren und bekannten Anwendung der verwendeten Präparate und der MR-Diagnostik ausgegangen werden.

Die Auswertung toxikologischer, pharmakologischer oder sicherheitsrelevanter, technischer Parameter der im Rahmen der Routinebehandlung der Patienten eingesetzten Arzneimittel und Medizinprodukte sind nicht Teil dieser Studie. Aus diesem Grund werden in dieser Studie keine unerwünschten Ereignisse (Adverse Events, AEs) oder unerwünschten Medizinproduktreaktionen (adverse device effect, ADEs) definiert und aufgezeichnet.

# 8. Biometrie

## 8.1 Endpunkte

Der primäre Endpunkt ist das Ansprechen auf die neoadjuvante Therapie. Das Ansprechen wird mittels histologischer Untersuchung im Resektat (pTNM) und TRG [24] [25] ermittelt. Ein Ansprechen liegt bei einem TRG-Score von 1a/b oder 2 vor. Ein Nichtansprechen liegt bei einem TRG-Score von 3 vor.

Sekundäre Endpunkte umfassen Veränderungen des Tumorvolumens und des Signales der einzelnen MR-Sequenzen, sowie eine differenziertere Betrachtung des Ansprechens auf die Therapie (ordinaler TRG-Score, Komplettremission). Die Rate von TILs mit ihren Subgruppen im Resektat sowie die Nachweisbarkeit bzw. Zahl von CTCs im Therapieverlauf sind weitere Endpunkte, die betrachtet werden. Ferner sind demografische Daten sowie Fragebögen zur Lebensqualität (EORTC QLQ-C30 / QLQ-CR29) und therapiebedingte Nebenwirkungen (erkrankungsbezogen entsprechend EORTC-CTCAE[8]) Gegenstand explorativer Analysen. Die Lokalrezidivrate, die Rate an Fernmetastasen sowie das Überleben jeweils nach 5 Jahren werden ebenfalls deskriptiv betrachtet.

## 8.2 Definition von Auswertungskollektiven

Es gibt drei Auswertungskollektive. (1) Das Gesamtkollektiv umfasst alle Studienteilnehmer. (2) Die Analysegruppe I umfasst die Studienteilnehmer, die eine vollständige konsolidierende Chemotherapie mit 8 oder 9 Zyklen FOLFOX4 erhalten. (3) Die Analysegruppe II besteht aus Studienteilnehmern, bei denen keine (vollständige) konsolidierende Chemotherapie möglich ist.

## 8.3 Planung des Studienumfanges (Fallzahlplanung)

Die Rationale der Patientenzahl leitet sich von der Machbarkeit ab, sodass eine Umsetzung einer Fallzahlplanung nicht möglich ist und das Ziel dieser Pilotstudie das Schätzen und nicht das Testen ist. Auf Basis der Eckdaten für die Durchführung der Behandlung ist ein Rekrutierungszeitraum von zwei Jahren möglich. Auf Basis vorliegender Fallzahlen im Studienzentrum werden in diesem Zeitraum 50 Patienten mit lokal fortgeschrittenem Rektumkarzinom (UICC-Stadium II/III) erwartet, von denen voraussichtlich 35 eine konsolidierende Chemotherapie erhalten werden (Analysegruppe I).

Die primäre Analyse ist die Erhebung der prädiktiven Wertigkeit der MRT-Parameter zur Vorhersage eines Ansprechens auf die neoadjuvante Therapie. Somit erfolgt diese Auswertung in der Analysegruppe I. Nimmt man auf Grundlage vorliegender Daten [5-7] und den bisherigen Fallzahlen unserer Klinik eine Ansprechrate von 60% an, so werden 30 der 50 Studienteilnehmer auf diese Therapie ansprechen. Damit ergibt sich, dass man eine Fläche unter der ROC-Kurve (AUC) von 0,72 mit einer statistischen Power von 0,80 zu einem Signifikanzniveau von 0,05 nachweisen kann. In Abbildung 2 werden nachweisbare AUCs für weitere Parameterkonstellationen dargestellt. Diese Berechnung erfolge mit R (Version 4.04) und dem R-Paket pROC (Version 1.18.0).

[8] CTC – common toxicity criteria analog EORTC

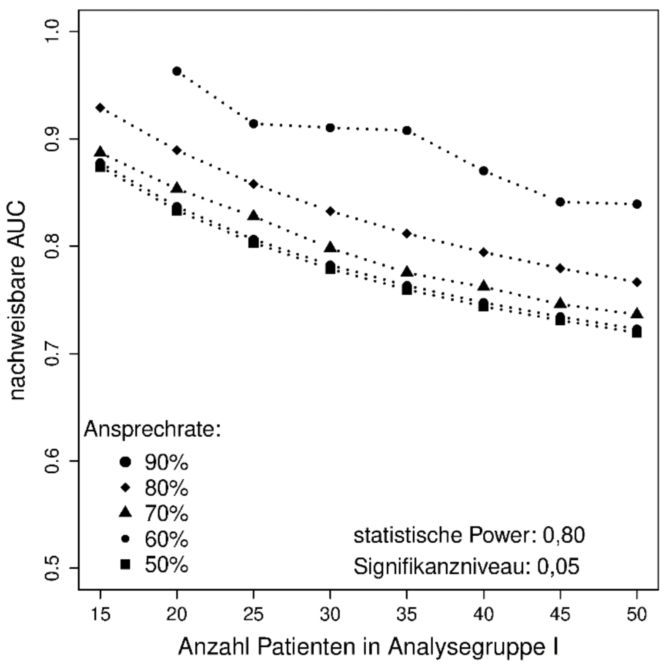


**Abbildung 2**. Nachweisbare Fläche unter der ROC-Kurve (AUC) für gegebene Anzahl an Patienten mit vollständiger TNT (Analysegruppe I) und Rate an Patienten, die auf die Therapie ansprechen. Abkürzungen: ROC, engl. receiver operating characteristic.

## 8.4 Zwischenauswertung(en)

Es werden keine Zwischenauswertungen durchgeführt.

Das Tumoransprechen wird histologisch am Operationspräparat nach Tumorresektion abschließend bewertet (Erreichen primärer Endpunkt), die Auswertung folgt daher in Q3/2025. Die abschließende Auswertung der sekundären Endpunkte erfolgt nach deren Erreichen im Rahmen der 5-Jahres-Nachsorge im Sinne einer gestaffelten Auswertung (V22).

## 8.5 Statistische Analyse

Die gesamte Auswertung ist explorativ. 95%-Konfidenzintervalle und p-Werte dienen der Beschreibung und keiner konfirmatorischen Schlussfolgerung.

Die primäre Auswertung des primären Endpunktes (Therapieansprechen) erfolgt in der Analysegruppe I. Die prädiktive Wertigkeit von DWI-Parametern auf das Therapieansprechen wird mittels AUC mit 95%-Konfidenzintervall quantifiziert. Als Surrogatmarker für das Therapieansprechen wird dabei die Veränderung der DWI-Parameter vom 1. zum 5. MRT betrachtet. Es wird die Identifikation des optimalen Trennwertes gemäß Youden-Index angestrebt. Im Anschluss erfolgt die deskriptive Auswertung des Therapieansprechens und die Beschreibung des Verlaufs der DWI-Parameter vom 1. bis zum 5. MRT. Zur Berücksichtigung der unterschiedlichen Quellen für die Erhebung des primären Endpunktes, welche durch das „watch and wait"-Konzept gegeben wird, erfolgt eine Sensitivitätsanalyse. Hierbei werden die Patienten mit bioptischer Sicherung der Tumorregression und die Patienten, bei denen eine Tumorresektion durchgeführt wurde, getrennt ausgewertet (AUC-Auswertung; deskriptive Beschreibung). In einer sekundären Auswertung wird die Veränderung vom 1. bis zum 3. MRT untersucht. Hierbei werden alle drei Auswertungskollektive betrachtet.

Für die Auswertung der sekundären Endpunkte kommen geeignete Methoden der beschreibenden und schließenden Statistik (absolute und relative Häufigkeiten; Median mit ersten und dritten Quartil; AUC) zur Anwendung. Zeitliche Verläufe werden graphisch dargestellt. Vergleiche zwischen Analysegruppe I und Analysegruppe II finden im Gesamtkollektiv statt. Hierfür wird je nach Variablentyp der U-Test, der Kruskal-Wallis-Test oder der exakte Fisher-Test verwendet.

## 8.6 Präsentation der Ergebnisse

Die Ergebnispräsentation erfolgt anhand der STROBE-Kriterien für Beobachtungsstudien [31] sowie der TRIPOD Kriterien [32] für prognostische Fragestellungen. Die Auswertung orientiert sich im Ablauf an den CONSORT-Kriterien [33].

# 9. Datenmanagement

## 9.1 Patientenidentifikationsliste

Alle patientenbezogenen Daten werden in pseudonymisierter Form erfasst. Dazu wird ein nicht-sprechendes Pseudonym verwendet, aus welchem allein nicht auf die Identität des Patienten geschlossen werden kann.

Das Prüfzentrum führt eine Patientenidentifikationsliste, in der die Patientenidentifikationsnummern (Patienten-ID) mit den vollen Patientennamen der Teilnehmer, Patienten-ID und ggf. Geburtsdatum verbunden sind. Diese Liste dient der Möglichkeit der späteren Identifikation teilnehmender Personen. Sie wird absolut vertraulich behandelt und darf das Prüfzentrum nicht verlassen. Sie wird nach Studienende **mindestens zehn Jahre** zu archiviert.

Zusätzlich wird die Teilnahme betreffender Personen an der Studie in der jeweiligen Patientenakte vermerkt.

## 9.2 Liste der Verantwortlichkeiten

Es muss sichergestellt werden, dass jede Person, die für die Dokumentation im eCRF verantwortlich ist, identifiziert werden kann. Eine Liste mit Unterschrift und Kürzel der Personen, die Eintragungen im eCRF vornehmen dürfen (Signature/Delegation Log), wird im ISF und im TMF abgelegt. Mit dieser Übersicht werden auch weitere Personen, die in die klinische Prüfung eingebunden sind, mit ihren Namen, Unterschrift und Kürzel sowie ihren Verantwortlichkeiten und Befugnissen benannt

## 9.3 Datenerhebung/Datenverarbeitung

Die Datenerfassung dient wissenschaftlichen Zwecken. Zur Erreichung des Studienzieles ist es erforderlich, medizinische Daten einzelner Patienten zu erheben und zu verarbeiten.

Alle für diese Studie erforderlichen Daten werden nach Diagnosestellung und Einwilligung des Patienten in Form von elektronischen CRFs (electronic Case Report Forms; eCRF) durch das Prüfzentrum in eine Remote Data Entry (RDE)-Datenbank eingegeben. Hierfür wird die browserbasierte Software „REDCap" genutzt. Die Speicherung der Daten erfolgt auf ausschließlich intern zugänglichen Servern des Universitätsklinikum Jena. Die personenbezogenen Daten eines eingeschlossenen Patienten werden im Zuge eines Pseudonymisierungverfahrens (siehe Kapitel 9.1) einer individuellen Identifikationsnummer (Patienten-ID) zugeordnet. Die Dokumentation klinischer Daten erfolgt ausschließlich über die vergebene Patienten-ID. Es liegt in der Verantwortung des Studienleiters, dass alle im Rahmen der Studie erhobenen Daten korrekt und vollständig in die speziell für diese erstellte Datenbank eingetragen werden. Korrekturen im eCRF dürfen nur von autorisierten Personen vorgenommen werden und sind zu begründen.

## 9.4 Aufbewahrung der Studienunterlagen (Archivierung)

Der Studienleiter muss sicherstellen, dass die wichtigsten Unterlagen für mindestens 10 Jahre nach Beendigung der Studie aufbewahrt werden. Wichtige Dokumente im Zusammenhang mit der Studie sind z.B. die Patientenidentifikationsliste, Einwilligungserklärungen, Korrespondenz mit der Ethik-Kommission, der Studienleitung und andere relevante Dokumente. Andere Vorschriften für die Aufbewahrung von medizinischen Unterlagen bleiben unberührt. Alle Unterlagen müssen an einem sicheren Ort aufbewahrt und vertraulich behandelt werden. Falls erforderlich (z. B. aufgrund gesetzlicher Bestimmungen oder nach Rücksprache mit dem Studienleiter) können die Unterlagen über den oben genannten Zeitraum hinaus aufgehoben werden. Das Studienzentrum muss Vorkehrungen treffen, um eine versehentliche oder vorzeitige Zerstörung dieser Dokumente zu verhindern

## 9.5 Datenschutz

Im Rahmen der Studie werden demographischen Daten der Studienteilnehmer (z. B. vollständiger Name, Initialen des Vor- und Zunamens, Geburtsdatum, Adresse) und Daten zur Behandlung und zum Krankheitsverlauf (z. B. medizinische Befunde, Behandlungsarten, verordnete Medikamente) erhoben und verarbeitet. Diese demographischen Daten sowie studienspezifisch erhobene, personenbezogene Daten und Dokumente, wie die Patienten-Identifikationsliste, die Patienteninformation und Einwilligungserklärung, Korrespondenz mit der Ethikkommission, werden zehn Jahre nach Studienende aufbewahrt (oder länger, wenn gesetzlich gefordert).
Die studienbedingt erhobenen, personenbezogenen Bildinformationen (MR-Datensätze) werden auf lokalen Laufwerken gespeichert (digitales, klinikinternes Bildarchiv). Aus diesen werden numerische Variablen entsprechend der definierten Messgrößen generiert, die pseudonymisiert im o.g. EDV-gestützten Online-Datenerfassungssystem gespeichert werden.

Alle erhobenen medizinischen Daten werden im Prüfzentrum mit Hilfe des o.g. EDV-gestützten Online-Datenerfassungssystems pseudonymisiert eingegeben und unmittelbar auf ein intern zugängliches Server-Laufwerk des Universitätsklinikums Jena übermittelt.

Der Schutz der personenbezogenen Studiendaten vor dem Zugriff Dritter wird durch folgende Maßnahmen gewährleistet:

- Klinische Angaben werden ausschließlich pseudonymisiert in die Datenbank aufgenommen, verarbeitet und verwaltet. Es werden keine Merkmale in die Datenbank übertragen, welche eine unmittelbare Identifizierung bestimmter Patienten erlauben.
- Bei der Dateneingabe, welche in der Klinik für Strahlentherapie erfolgt, werden die Bestimmungen des Datenschutzgesetzes eingehalten. Zugriff auf alle Daten der klinischen Studie haben nur autorisierte Personen des Prüfzentrums (siehe 9.2).
- Die Datenverarbeitung und –auswertung erfolgt anhand der pseudonymisierten Daten durch die Studienleitung in der Klinik für Strahlentherapie sowie durch die Biometrikerin am Institut für Medizinische Statistik, Informatik und Datenwissenschaften in Jena.
- Alle beteiligten Personen sind zur Verschwiegenheit verpflichtet.
- Die Daten sind vor fremden Zugriff geschützt.
- Ergebnisse von Auswertungen werden in anonymisierter Form publiziert, so dass eine Zuordnung von Daten zu individuellen Patienten nicht möglich ist.

# 10. Ethische Belange und administrative Regelungen

## 10.1 Deklaration von Helsinki und Gute klinische Praxis

Die Studie wird gemäß den ethischen Grundsätzen durchgeführt, die ihren Ursprung in der Deklaration von Helsinki haben. Die jeweils aktuelle Version der Deklaration wird beachtet.

Die Empfehlungen der Guten Klinischen Praxis, gültig seit dem 17.1.1997, werden, sofern zutreffend, berücksichtigt.

## 10.2 Ethik-Kommissionen

Das Studienprotokoll wird mit den erforderlichen weiteren Unterlagen der zuständigen federführenden Ethik-Kommission des Studienleiters mit der Bitte um Bewertung vorgelegt. Die Studie kann erst nach zustimmender Bewertung der Ethik-Kommission beginnen.

## 10.3 Nachträgliche Änderungen

Das Studienprotokoll ist einzuhalten. Jede vom Prüfer zu vertretende Abweichung von den vorgesehenen Untersuchungs- und Behandlungsmaßnahmen oder -zeitpunkten ist zu dokumentieren und zu begründen.

Änderungen oder Ergänzungen des Studienprotokolls können nur von der Studienleitung veranlasst und autorisiert werden. Über Änderungen des Studienprotokolls werden die federführende Ethikkommission und die Ethik-Kommissionen der beteiligten Prüfzentren informiert. Ggf. wird erneut die zustimmende Bewertung eingeholt. Bewertungspflichtige Änderungen dürfen nicht vor der Entscheidung der Ethikkommission umgesetzt werden.

Änderungen der von der Ethik-Kommission zustimmend bewerteten Studie, die geeignet sind, sich auf die Sicherheit der betroffenen Personen auszuwirken, bei zusätzlichen Datenerhebungen oder Auswertungen, die eine Änderung der Patienteninformation und/oder -einwilligung erfordern, bei der Auslegung der wissenschaftlichen Dokumente, auf die die Studie gestützt wird, oder die die wissenschaftliche Aussagekraft der Studienergebnisse zu beeinflussen, die die Art der Leitung oder Durchführung der Studie wesentlich zu verändern, dürfen nur vorgenommen werden, wenn diese Änderungen von der Ethik-Kommission zustimmend bewertet wurden.

## 10.4 Registrierung

Die vorliegende Studie wird vor Einschluss des ersten Studienpatienten im Register *clinicaltrials.gov* registriert.

## 10.5 Finanzierung

Das wird im Rahmen des IZKF *„clinician scientist“* Programm für 3 Jahre gefördert. Im Rahmen dessen stehen projektbezogene Drittmittel zur Verfügung. Zudem wurden Drittmittel im Rahmen des *„Life Talent Funds“* der Friedrich-Schiller-Universität Jena / Profillinie „LIFE“ eingeworben.

Weitere, davon nicht gedeckte Posten werden durch Hausmittel der Klinik für Strahlentherapie und Radioonkologie finanziert.

## 10.6 Abschlussbericht und Publikation

Es ist geplant, das finale konsentierte Studienprotokoll gemäß den Gepflogenheiten internationaler Studiengruppen und den unten aufgeführten Regularien in einem Journal mit Peer Review zu publizieren.

Die Veröffentlichung der Studienergebnisse erfolgt unabhängig davon, wie die Ergebnisse ausfallen.

# 11. Anlagen

## 11.1 Anlage 1 Regressions-Skala (TRG) nach TU München

(Becker et al.[25] / Werner und Höfler [24])

| **Regressionsgrading nach Becker et al**. (Cancer 2003, 98: 1521-1530.)[25] | | |
|---|---|---|
| **1a** | Complete response (cR) | Keine Tumorzellen nachweisbar |
| **1b** | Subtotal response (sR) | <10% vitale Tumorzellen |
| **2** | Partielle Response (PR) | 10-50% vitale Tumorzellen |
| **3** | Minimale Response (MR) | >50% vitale Tumorzellen |
| | Keine Response (NR) | Keine histologischen Regressionszeichen |

## 11.2 Anlage 2: Lebensqualitätfragebogen EORTC QLQ-C30

GERMAN

### EORTC QLQ-C30 (Version 3)

Wir sind an einigen Angaben interessiert, die Sie und Ihre Gesundheit betreffen. Bitte beantworten Sie die folgenden Fragen selbst, indem Sie die Zahl einkreisen, die am besten auf Sie zutrifft. Es gibt keine „richtigen“ oder „falschen“ Antworten. Ihre Angaben werden streng vertraulich behandelt.

Bitte tragen Sie Ihre Initialen ein: |_|_|_|_|

Ihr Geburtsdatum (Tag, Monat, Jahr): |_|_|_|_|_|_|_|_|

Das heutige Datum (Tag, Monat, Jahr): 31 |_|_|_|_|_|_|_|_|

---

| | Überhaupt nicht | Wenig | Mäßig | Sehr |
|---|---|---|---|---|
| 1. Bereitet es Ihnen Schwierigkeiten, sich körperlich anzustrengen (z. B. eine schwere Einkaufstasche oder einen Koffer zu tragen)? | 1 | 2 | 3 | 4 |
| 2. Bereitet es Ihnen Schwierigkeiten, einen <u>längeren</u> Spaziergang zu machen? | 1 | 2 | 3 | 4 |
| 3. Bereitet es Ihnen Schwierigkeiten, eine <u>kurze</u> Strecke außer Haus zu gehen? | 1 | 2 | 3 | 4 |
| 4. Müssen Sie tagsüber im Bett liegen oder in einem Sessel sitzen? | 1 | 2 | 3 | 4 |
| 5. Brauchen Sie Hilfe beim Essen, Anziehen, Waschen oder Benutzen der Toilette? | 1 | 2 | 3 | 4 |

**Während der letzten Woche:**

| | Überhaupt nicht | Wenig | Mäßig | Sehr |
|---|---|---|---|---|
| 6. Waren Sie bei Ihrer Arbeit oder bei anderen tagtäglichen Beschäftigungen eingeschränkt? | 1 | 2 | 3 | 4 |
| 7. Waren Sie bei Ihren Hobbys oder anderen Freizeitbeschäftigungen eingeschränkt? | 1 | 2 | 3 | 4 |
| 8. Waren Sie kurzatmig? | 1 | 2 | 3 | 4 |
| 9. Hatten Sie Schmerzen? | 1 | 2 | 3 | 4 |
| 10. Mussten Sie sich ausruhen? | 1 | 2 | 3 | 4 |
| 11. Hatten Sie Schlafstörungen? | 1 | 2 | 3 | 4 |
| 12. Fühlten Sie sich schwach? | 1 | 2 | 3 | 4 |
| 13. Hatten Sie Appetitmangel? | 1 | 2 | 3 | 4 |
| 14. War Ihnen übel? | 1 | 2 | 3 | 4 |
| 15. Haben Sie erbrochen? | 1 | 2 | 3 | 4 |
| 16. Hatten Sie Verstopfung? | 1 | 2 | 3 | 4 |

<u>Bitte wenden</u>

GERMAN

| **Während der letzten Woche:** | **Überhaupt nicht** | **Wenig** | **Mäßig** | **Sehr** |
|---|---|---|---|---|
| 17. Hatten Sie Durchfall? | 1 | 2 | 3 | 4 |
| 18. Waren Sie müde? | 1 | 2 | 3 | 4 |
| 19. Fühlten Sie sich durch Schmerzen in Ihrem alltäglichen Leben beeinträchtigt? | 1 | 2 | 3 | 4 |
| 20. Hatten Sie Schwierigkeiten, sich auf etwas zu konzentrieren, z. B. auf Zeitunglesen oder Fernsehen? | 1 | 2 | 3 | 4 |
| 21. Fühlten Sie sich angespannt? | 1 | 2 | 3 | 4 |
| 22. Haben Sie sich Sorgen gemacht? | 1 | 2 | 3 | 4 |
| 23. Waren Sie reizbar? | 1 | 2 | 3 | 4 |
| 24. Fühlten Sie sich niedergeschlagen? | 1 | 2 | 3 | 4 |
| 25. Hatten Sie Schwierigkeiten, sich an Dinge zu erinnern? | 1 | 2 | 3 | 4 |
| 26. Hat Ihr körperlicher Zustand oder Ihre medizinische Behandlung Ihr Familienleben beeinträchtigt? | 1 | 2 | 3 | 4 |
| 27. Hat Ihr körperlicher Zustand oder Ihre medizinische Behandlung Ihr Zusammensein oder Ihre gemeinsamen Unternehmungen mit anderen Menschen beeinträchtigt? | 1 | 2 | 3 | 4 |
| 28. Hat Ihr körperlicher Zustand oder Ihre medizinische Behandlung für Sie finanzielle Schwierigkeiten mit sich gebracht? | 1 | 2 | 3 | 4 |

**Bitte kreisen Sie bei den folgenden Fragen die Zahl zwischen 1 und 7 ein, die am besten auf Sie zutrifft:**

29. Wie würden Sie insgesamt Ihren Gesundheitszustand während der letzten Woche einschätzen?

| 1 | 2 | 3 | 4 | 5 | 6 | 7 |
|---|---|---|---|---|---|---|
| sehr schlecht | | | | | | ausgezeichnet |

30. Wie würden Sie insgesamt Ihre Lebensqualität während der letzten Woche einschätzen?

| 1 | 2 | 3 | 4 | 5 | 6 | 7 |
|---|---|---|---|---|---|---|
| sehr schlecht | | | | | | ausgezeichnet |

© QLQ-C30 Copyright 1995 EORTC Quality of Life Group. Alle Rechte vorbehalten. Version 3.0

## 11.3 Anlage 3: Lebensqualitätfragebogen EORTC QLQ-CR29

### EORTC QLQ – CR29

Patienten berichten manchmal die nachfolgend beschriebenen Symptome oder Probleme. Bitte beschreiben Sie, wie stark Sie diese Symptome oder Probleme während der letzten Woche empfunden haben. Kreuzen Sie bitte die Zahl an, die am ehesten auf Sie zutrifft.

| Während der letzten Woche: | Überhaupt nicht | Wenig | Mäßig | Sehr |
|---|---|---|---|---|
| 31. Mussten Sie tagsüber häufig Wasser lassen? | 1 | 2 | 3 | 4 |
| 32. Mussten Sie nachts häufig Wasser lassen? | 1 | 2 | 3 | 4 |
| 33. Kam es bei Ihnen zu unwillkürlichem Harnabgang? | 1 | 2 | 3 | 4 |
| 34. Hatten Sie Schmerzen beim Wasserlassen? | 1 | 2 | 3 | 4 |
| 35. Hatten Sie Bauchschmerzen? | 1 | 2 | 3 | 4 |
| 36. Hatten Sie Schmerzen im Gesäß/Analbereich/Rektum? | 1 | 2 | 3 | 4 |
| 37. Hatten Sie das Gefühl, dass Ihr Bauch gebläht ist? | 1 | 2 | 3 | 4 |
| 38. Hatten Sie Blut im Stuhl? | 1 | 2 | 3 | 4 |
| 39. Befand sich Schleim in Ihrem Stuhlgang? | 1 | 2 | 3 | 4 |
| 40. Hatten Sie einen trockenen Mund? | 1 | 2 | 3 | 4 |
| 41. Hatten Sie aufgrund Ihrer Behandlung Haarausfall? | 1 | 2 | 3 | 4 |
| 42 Hatten Sie Probleme mit Ihrem Geschmackssinn? | 1 | 2 | 3 | 4 |

| Während der letzten Woche: | Überhaupt nicht | Wenig | Mäßig | Sehr |
|---|---|---|---|---|
| 43. Waren Sie wegen Ihres zukünftigen Gesundheitszustandes besorgt? | 1 | 2 | 3 | 4 |
| 44. Haben Sie sich Sorgen über Ihr Gewicht gemacht? | 1 | 2 | 3 | 4 |
| 45. Fühlten Sie sich wegen Ihrer Erkrankung oder Behandlung körperlich weniger anziehend? | 1 | 2 | 3 | 4 |
| 46. Fühlten Sie sich wegen Ihrer Erkrankung oder Behandlung weniger weiblich/männlich? | 1 | 2 | 3 | 4 |
| 47. Waren Sie mit Ihrem Körper unzufrieden? | 1 | 2 | 3 | 4 |
| 48. Haben Sie ein Stoma (künstlicher Darmausgang)? (Zutreffendes bitte ankreuzen) | ja | | nein | |

Fortsetzung auf der nächsten Seite

## Während der letzten Woche:

**Beantworten Sie die folgenden Fragen bitte NUR, WENN SIE EINEN STOMABEUTEL TRAGEN. Fahren Sie ansonsten weiter unten fort:**

| | Überhaupt nicht | Wenig | Mäßig | Sehr |
|---|---|---|---|---|
| 49. Hatten Sie unfreiwillige Darmgasentweichungen/Flatulenzen aus Ihrem Stomabeutel? | 1 | 2 | 3 | 4 |
| 50. Hatten Sie ungewollte Stuhlabgänge aus Ihrem Stomabeutel? | 1 | 2 | 3 | 4 |
| 51. War die Haut um Ihr Stoma wund? | 1 | 2 | 3 | 4 |
| 52. Gab es im Tagesverlauf häufige Beutelwechsel? | 1 | 2 | 3 | 4 |
| 53. Fanden während der Nacht häufige Beutelwechsel statt? | 1 | 2 | 3 | 4 |
| 54. War es Ihnen peinlich, ein Stoma zu haben? | 1 | 2 | 3 | 4 |
| 55. Hatten Sie Probleme mit Ihrer Stomapflege? | 1 | 2 | 3 | 4 |

**Beantworten Sie die folgenden Fragen NUR, WENN SIE KEINEN STOMABEUTEL TRAGEN:**

| | Überhaupt nicht | Wenig | Mäßig | Sehr |
|---|---|---|---|---|
| 49. Hatten Sie unfreiwillige Darmgasentweichungen/Flatulenzen aus Ihrem Darmausgang? | 1 | 2 | 3 | 4 |
| 50. Hatten Sie ungewollte Stuhlabgänge aus Ihrem Darmausgang? | 1 | 2 | 3 | 4 |
| 51. Hatten Sie wunde Haut in Ihrem Analbereich? | 1 | 2 | 3 | 4 |
| 52. Gab es im Tagesverlauf häufige Stuhlgänge? | 1 | 2 | 3 | 4 |
| 53. Gab es während der Nacht häufige Stuhlgänge? | 1 | 2 | 3 | 4 |
| 54. Waren Ihnen die Stuhlgänge peinlich? | 1 | 2 | 3 | 4 |

## Während der letzten 4 Wochen:

| **Nur für Männer:** | Überhaupt nicht | Wenig | Mäßig | Sehr |
|---|---|---|---|---|
| 56. Wie sehr waren Sie an Sexualität interessiert? | 1 | 2 | 3 | 4 |
| 57. Hatten Sie Schwierigkeiten, eine Erektion zu bekommen oder zu halten? | 1 | 2 | 3 | 4 |

| **Nur für Frauen:** | Überhaupt nicht | Wenig | Mäßig | Sehr |
|---|---|---|---|---|
| 58. Wie sehr waren Sie an Sexualität interessiert? | 1 | 2 | 3 | 4 |
| 59. Verspürten Sie Schmerzen oder Beschwerden während des Geschlechtsverkehrs? | 1 | 2 | 3 | 4 |

© Copyright 2006 EORTC QLQ-CR29 Quality of Life Group. All rights reserved. Alle Rechte vorbehalten. Version 2.1

## 11.4 Anlage 4: Fragebogen zur Erfassung therapiebedingter Nebenwirkungen

adaptiert nach EORTC CTCAE v5.0 (2017)

UNIVERSITÄTS KLINIKUM jena

**Klinik für Strahlentherapie**
Direktorin: Prof. Dr. A. Wittig

Zentrum für ambulante Medizin
Uniklinikum Jena

**Untersuchungsbefund**

**Vorstellung Becken**

vor Strahlentherapie Datum: {Today}

{Patient.Salutation}
{Patient.Last_Name},
{Patient.First_Name}
{Admin.Birth_Date} ({Admin.Age}),
{Admin.Gender}
{Admin.Pat_Adr1} - {Admin.Pat_Postal} - {Admin.Pat_City}
Mosaiq-ID: {Ident.IDA}

**(Zwischen-) anamnese:**

**Aktuelle Beschwerden (weiteres siehe CTC-Bogen):**

**Medikation / Systemtherapie:**

**Gewicht:** kg konstant ( kg - in Monaten) **Größe:** cm **ECOG/KI:**

**Letztes Staging am:** Befund:

| | Grad 0 | Grad I | Grad II | Grad III | Grad IV |
|---|---|---|---|---|---|
| **Allgemein** | | | | | |
| **Fatigue** | ☐ keine | ☐ Fatique, durch Ruhe erleichtert | ☐ Fatigue, durch Ruhe nicht erleichtert; Einschränkung der instrumentellen ATL | ☐ Fatigue; durch Ruhe nicht erleichtert; eingeschränkte Selbstversorgung | - |
| **Malaise / Krankheitsgefühl** | ☐ keine | ☐ Unbehagen oder Fehlen des Wohlseins | ☐ Unbehagen oder Fehlen des Wohlseins; begrenzte instrumentelle ATL | - | - |
| **Schmerz** | ☐ keine | ☐ Leichter Schmerz | ☐ Mäßiger Schmerz; Einschränkung der instrumentellen ATL | ☐ Schwerer Schmerz; Einschränkung der selbstversorgenden ATL | - |
| **Strahlendermatitis** | ☐ keine | ☐ Schwache Rötung oder trockene Hautschuppung | ☐ Mäßiges bis ausgeprägtes Erythem; ungleichmäßige feuchte Desquamation, überwiegend auf Hautfalten beschränkt; mäßiges Ödem | ☐ Feuchte Desquamation außerhalb von Hautfalten, erhöhte Blutungsneigung durch geringes Trauma oder Abschürfung | ☐ Lebensbedrohliche Auswirkungen; Hautnekrose oder Ulzeration der gesamten Kutis spontane Blutungen des betroffenen Gebietes; Hauttransplantation indiziert |
| **GI Trakt** | | | | | |
| **Diarrhoe** | ☐ keine | ☐ Erhöhung <4 Stühle pro Tag über Normalfrequenz; geringer Anstieg der Stuhlmenge über die Normal-menge hinaus | ☐ Erhöhung 4-6 Stühle pro Tag über die Normal-frequenz hinaus; mäßiger Anstieg der Stuhlmenge über die Normalmenge hinaus | ☐ Erhöhung >= 7 Stühle pro Tag über die Normalfrequenz hinaus; Inkontinenz; Krankenhauseinweisung angezeigt; schwerwiegender Anstieg der Stuhlmenge über die Normalmenge hinaus; Einschränkung der selbstversorgenden | ☐ Lebensbedrohliche Folgen; Intervention dringlich angezeigt |
| **Opstipation** | ☐ keine | ☐ gelegentl. o. intermitt. Sympt.; gelegentlich stuhlerweich. Med. / Laxantien/Klistier/ Diät-Modifizierung | ☐ keine dauerhafte Sympt. mit regelmäß. Gebrauch von Laxan-tien/Klistieren; EIA | ☐ Obstipation mit Indikation zur manuellen Ausräumung; EAF | ☐ Lebensbedrohliche Folgen; Intervention dringlich angezeigt. |
| **Rektale Blutung** | ☐ keine | ☐ Leicht; Intervention nicht angezeigt | ☐ Mäßige Symptome; medizinische Intervention oder geringfügige Kauterisation angezeigt | ☐ Transfusion; radiologische, endoskopische oder elektive operative Intervention angezeigt | ☐ Lebensbedrohliche Folgen; Intervention dringlich angezeigt. |
| **Proktitis** | ☐ keine | ☐ Rektale Beschwerden; Intervention nicht angezeigt. | ☐ Symptome (z. B. rektale Beschwerden, Blut oder Schleim im Stuhl ); medizinische Intervention angezeigt; Einschränkung der instrumentellen ATL | ☐ Schwere Symptome; Stuhldrang oder Stuhlinkontinenz; begrenzte selbstversorgende ATL | ☐ Lebensbedrohliche Folgen; Intervention dringlich angezeigt. |
| **Ileus** | ☐ kein | ☐ kein | ☐ symptomatisch, veränderte GI-Funktion; Darmruhe angezeigt | ☐ stark veränderte GI-Funktion; TPN indiziert | ☐ lebensbedrohlich; dringende Intervention indiziert |
| **Stuhlinkontinenz** | ☐ keine | ☐ Gelegentlicher Gebrauch von Windeln erforderlich | ☐ Täglicher Gebrauch von Windeln erforderlich | ☐ Schwere Symptome; elektive operative Intervention angezeigt | ☐ Lebensbedrohliche Folgen; Intervention dringlich angezeigt |

verantwortlich: Dr. S. Drozdz Stand: 12.03.2021 Dokument1
UK Jena - Klinik für Strahlentherapie und Radioonkologie Seite 1 von 2

UNIVERSITÄTSKLINIKUM jena

**Klinik für Strahlentherapie**
Direktorin: Prof. Dr. A. Wittig

Zentrum für ambulante Medizin
Uniklinikum Jena

{Patient.NameLFM} geb.:{Admin.Birth_Date} ID: {Ident.IDA}

| | | | | | |
|---|---|---|---|---|---|
| **GI-Fistel** | ☐ keine | ☐ Asymptomatisch; lediglich klinische oder diagnostische Beobachtung; Intervention nicht angezeigt | ☐ Symptomatisch; veränderte gastrointestinale Funktion | ☐ Stark veränderte gastrointestinale Funktion; Sondenernährung; TPE oder Krankenhauseinweisung angezeigt | ☐ Lebensbedrohliche Auswirkungen; Intervention dringend angezeigt |
| **GU-Trakt** | | | | | |
| **Inkontinenz** | ☐ keine | ☐ Gelegentlich (z. B. beim Husten, Niesen, etc.), Vorlagen (Binden) nicht angezeigt | ☐ Spontan; Vorlagen (Binden) angezeigt; Einschränkung der instrumentellen ATL | ☐ Intervention angezeigt (z. B. Klemme, Kollageninjektionen); operative Intervention angezeigt; Einschränkung der selbstversorgenden ATL | - |
| **Cystitis** | ☐ keine | ☐ mikr. Hämaturie; min. Steigerung d. Häufigkeit, Dringlichkeit, Dysurie oder Nykturie; neuer Beginn der Inkontinenz | ☐ mod. Hämaturie; mod. Steigerung d. Häufigkeit, Dringlichkeit, Dysurie, Nykturie oder Inkonti-enz; neuer Beginn der Inkont-nenz; Urinkath. o. Blasenspülung notw.; EIA | ☐ mass. Hämaturie; Transfusion, iv-Med. oder Hospitalisierung erforderlich; elektive Endoskopie, radiolog. oder operative Interv. notw. | ☐ Lebensbedrohliche Auswirkungen; Intervention dringend angezeigt |
| **Vermehrter Harndrang** | ☐ keine | ☐ Vorhanden | ☐ Mäßiger Schmerz; Einschränkung der instrumentellen ATL | ☐ Schwerer Schmerz; Einschränkung der selbstversorgenden ATL | - |
| **Hämaturie** | ☐ keine | ☐ Asymptomatisch; lediglich klinische oder diagnostische Beobachtung; Intervention nicht angezeigt | ☐ Symptomatisch; Urinkathether oder Blasenspülung angezeigt; Einschränkung der instrumentellen ATL | ☐ Makroskopische Hämaturie; Transfusion, i. v. Medikationen oder Krankenhauseinweisung angezeigt; elektive endoskopische, radiologische oder operative Intervention angezeigt; Einschränkung der selbstversorgenden ATL | ☐ Lebensbedrohliche Auswirkungen; dringende radiologische oder operative Intervention angezeigt |
| **Harnstau** | ☐ keine | ☐ Setzen eines Urin-, suprapubischen oder intermittierenden Katheters nicht angezeigt; fähig mit einigem Restharn zu entleeren | ☐ Setzen eines Urin-, suprapubischen oder intermittierenden Katheters angezeigt; Medikation angezeigt | ☐ Elektive operative oder radiologische Intervention angezeigt; substantieller Verlust der betroffenen Nierenfunktion oder -masse | ☐ Lebensbedrohlich Organversagen; dringende operative Intervention angezeigt |
| **Vaginale Trockenheit** | ☐ keine | ☐ Leicht vaginale Trockenheit, die nicht mit sexueller Funktion interferiert | ☐ Mäßige vaginale Trockenheit, die mit sexueller Funktion interferiert oder häufige Beschwerden hervorruft | ☐ Schwere vaginale Trockenheit, die Dyspareunie oder schwerwiegende Beschwerden hervorruft | - |
| **Vaginale Stenose** | ☐ keine | ☐ Asymptomatisch; lediglich klinische oder diagnostische Beobachtung; Intervention nicht angezeigt | ☐ Vaginalverengung und/oder -verkürzung, die körperliche Untersuchung nicht beeinträchtigend | ☐ Vaginalverengung und/oder -verkürzung, den Gebrauch von Tampons, die sexuelle Aktivität oder die körperliche Untersuchung beeinträchtigend | - |
| **Erektile Dysfunktion** | ☐ keine | ☐ keine Herabsetzung der erektilen Funktion (Häufigkeit oder Rigidität der Erektionen), Intervention aber nicht angezeigt (z. B. Medikation oder Anwendung mechanische Mittel Penispumpe) | ☐ keine Herabsetzung der erektilen Funktion (Häufigkeit/Rigidität der Erektionen), erektile Intervention angezeigt (z. B. Medikation oder mechanische Mittel wie Penispumpe) | ☐ Herabsetzung der erektilen Funktion (Häufigkeit / Rigidität der Erektionen), aber erektile Intervention nicht hilfreich (z. B. Medikation oder mechanische Mittel wie Penispumpe); Setzen einer permanenten Penisprothese angezeigt (vorher nicht vorhanden) | - |
| **Becken** | | | | | |
| **Hüftfraktur / Hüftersatz** | ☐ keine | ☐ keine | ☐ Haarlinienfraktur; leichte Schmerzen; Einschränkung der instrumentellen ATL; nichtchirurgische Intervention angezeigt | ☐ Schwerer Schmerz; Krankenhauseinweisung zur Schmerzkontrolle angezeigt (z. B. Traktion); operative Intervention angezeigt | ☐ Lebensbedrohliche Auswirkungen; Symptome assoziiert mit neuro-vaskulärer Beeinträchtigung |
| **Lymphödem** | ☐ keine | ☐ Spuren von Verdickung oder leichte Verfärbung | ☐ Deutliche Verfärbung; lederartige Hauttextur; Papillenbildung; Einschränkung der instrumentellen ATL | ☐ Schwere Symptome; Einschränkung der selbstversorgenden ATL | - |
| **Mukositis (Anal, Vaginal)** | ☐ keine | ☐ Asymptomatisch oder geringe Symptome; Intervention nicht angezeigt | ☐ Symptomatisch; medizinische Intervention angezeigt; eingeschränkte altersgerechte instrumentelle ATL | ☐ Schwere Symptome; Einschränkung der selbstversorgenden ATL | ☐ Lebensbedrohliche Auswirkungen; Intervention dringend angezeigt |
| **Sonstiges:** | | | | | |

| Tumorstatus | unauffällig | suspekt | Befund | Befunddatum | Bemerkungen |
|---|---|---|---|---|---|
| kein Anhalt für Rezidiv/Progression ☐ | | | | | |
| Anal/Rektal | ☐ | ☐ | ☐ | | |
| Zervix | ☐ | ☐ | ☐ | | |
| Vaginal | ☐ | ☐ | ☐ | | |
| Vulva | ☐ | ☐ | ☐ | | |
| FM | ☐ | ☐ | ☐ | | |

**Weiterführende Diagnostik durch uns**

☐ keine ☐ BB ☐ S-Test ☐ TU-Marker ☐ Sono ☐ Skelettszintl. ☐ Mammographie ☐ Rö / CT-Thorax ☐ Sonstiges:

**Weiteres Vorgehen** **Datum:**

Wiedervorstellung in Monaten Unterschrift/Stempel Facharzt:

Datum Wiedervorstellung: Unterschrift/Stempel durchführender Arzt:

verantwortlich: Dr. S. Drozdz Stand: 12.03.2021 Dokument1
UK Jena - Klinik für Strahlentherapie und Radioonkologie Seite 2 von 2

## 11.5 **Anlage 5:** Karnofsky Performance Status (KPS)

(Karnofsky et al. [34])

| **Karnofsky Performance Status [%]** | |
|---|---|
| **100** | Normalzustand, keine Beschwerden keine Manifeste Erkrankung |
| **90** | Normale Leistungsfähigkeit, minimale Krankheitssymptome |
| **80** | Normale Leistungsfähigkeit mit Anstrengung, geringe Krankheitssymptome |
| **70** | Eingeschränkte Leistungsfähigkeit, arbeitsunfähig, kann sich selbst versorgen |
| **60** | Eingeschränkte Leistungsfähigkeit, benötigt gelegentlich fremde Hilfe |
| **50** | Eingeschränkte Leistungsfähigkeit, braucht krankenpflegerische und ärztliche Betreuung, nicht dauernd bettlägerig |
| **40** | Bettlägerig, spezielle Pflege erforderlich |
| **30** | Schwer krank, Krankenhauspflege notwendig |
| **20** | Schwer krank, Krankenhauspflege und supportive Maßnahmen erforderlich |
| **10** | Moribund, Krankheit schreitet schnell fort |
| **0** | Tod |

# 12. Literatur


1. DGHO. *Positionspapier Dihydropyrimidin-Dehydrogenase (DPD) -Testung vor Einsatz von 5-Fluorouracil, Capecitabin und Tegafur*. 2020 15.12.2021]; Available from: https://www.dgho.de/publikationen/stellungnahmen/gute-aerztliche-praxis/dpd-testung/dpd-positionspapier-2020-konsens_logos_final.pdf.
2. ZfKD. *Deutsches Krebsregister*. 2019 17/12/2021]; Available from: https://www.krebsdaten.de/Krebs/DE/Content/Krebsarten/Darmkrebs/darmkrebs_node.html.
3. Glynne-Jones, R., et al., *Rectal cancer: ESMO Clinical Practice Guidelines for diagnosis, treatment and follow-up†.* Annals of Oncology, 2017. **28**: p. iv22-iv40.
4. Sauer, R., et al., *Preoperative versus Postoperative Chemoradiotherapy for Rectal Cancer.* New England Journal of Medicine, 2004. **351**(17): p. 1731-1740.
5. Fokas, E., et al., *Randomized Phase II Trial of Chemoradiotherapy Plus Induction or Consolidation Chemotherapy as Total Neoadjuvant Therapy for Locally Advanced Rectal Cancer: CAO/ARO/AIO-12.* Journal of Clinical Oncology, 2019. **37**(34): p. 3212-3222.
6. Bahadoer, R.R., et al., *Short-course radiotherapy followed by chemotherapy before total mesorectal excision (TME) versus preoperative chemoradiotherapy, TME, and optional adjuvant chemotherapy in locally advanced rectal cancer (RAPIDO): a randomised, open-label, phase 3 trial.* The Lancet Oncology, 2021. **22**(1): p. 29-42.
7. Conroy, T., et al., *Neoadjuvant chemotherapy with FOLFIRINOX and preoperative chemoradiotherapy for patients with locally advanced rectal cancer (UNICANCER-PRODIGE 23): a multicentre, randomised, open-label, phase 3 trial.* The Lancet Oncology, 2021. **22**(5): p. 702-715.
8. Gani, C., A. Kirschniak, and D. Zips, *Watchful Waiting after Radiochemotherapy in Rectal Cancer: When Is It Feasible?* Visceral Medicine, 2019. **35**(2): p. 119-123.
9. Martens, M.H., et al., *Long-term Outcome of an Organ Preservation Program After Neoadjuvant Treatment for Rectal Cancer.* JNCI: Journal of the National Cancer Institute, 2016. **108**(12).
10. Burbach, J.P.M., et al., *Impact of radiotherapy boost on pathological complete response in patients with locally advanced rectal cancer: A systematic review and meta-analysis.* Radiotherapy and Oncology, 2014. **113**(1): p. 1-9.
11. Schurink, N.W., D.M.J. Lambregts, and R.G.H. Beets-Tan, *Diffusion-weighted imaging in rectal cancer: current applications and future perspectives.* The British journal of radiology, 2019. **92**(1096): p. 20180655-20180655.
12. Amodeo, S., et al., *MRI-Based Apparent Diffusion Coefficient for Predicting Pathologic Response of Rectal Cancer After Neoadjuvant Therapy: Systematic Review and Meta-Analysis.* American Journal of Roentgenology, 2018. **211**(5): p. W205-W216.
13. Kuremsky, J.G., J.E. Tepper, and H.L. McLeod, *Biomarkers for Response to Neoadjuvant Chemoradiation for Rectal Cancer.* International Journal of Radiation Oncology*Biology*Physics, 2009. **74**(3): p. 673-688.
14. Ryan, J.E., et al., *Predicting pathological complete response to neoadjuvant chemoradiotherapy in locally advanced rectal cancer: a systematic review.* Colorectal Dis, 2016. **18**(3): p. 234-46.
15. Salgado, R., et al., *The evaluation of tumor-infiltrating lymphocytes (TILs) in breast cancer: recommendations by an International TILs Working Group 2014.* Annals of oncology : official journal of the European Society for Medical Oncology, 2015. **26**(2): p. 259-271.
16. Kong, J.C., et al., *Prognostic Impact of Tumor-Infiltrating Lymphocytes in Primary and Metastatic Colorectal Cancer: A Systematic Review and Meta-analysis.* Dis Colon Rectum, 2019. **62**(4): p. 498-508.
17. Huang, Y., et al., *Local environment in biopsy better predict the pathological response to neoadjuvant chemoradiotherapy in rectal cancer.* Bioscience reports, 2019. **39**(3): p. BSR20190003.
18. Mirjolet, C., et al., *Tumor lymphocyte immune response to preoperative radiotherapy in locally advanced rectal cancer: The LYMPHOREC study.* Oncoimmunology, 2017. **7**(3): p. e1396402-e1396402.
19. Gold, M., et al., *Monitoring of circulating epithelial tumor cells using the Maintrac<sup>®</sup> method and its potential benefit for the treatment of patients with colorectal cancer.* Mol Clin Oncol, 2021. **15**(4): p. 201.
20. Asbach, P. and M. Taupitz, *Primäres MRT-Staging des Rektumkarzinoms*, in *MRT-basierte Chirurgie des Rektumkarzinoms*, M.E. Kreis and P. Asbach, Editors. 2020, Springer: Berlin, Heidelberg. p. 45-68.

21. Joye, I., et al., *The role of diffusion-weighted MRI and 18F-FDG PET/CT in the prediction of pathologic complete response after radiochemotherapy for rectal cancer: A systematic review.* Radiotherapy and Oncology, 2014. **113**(2): p. 158-165.
22. Her, E.J., et al., *Biologically Targeted Radiation Therapy: Incorporating Patient-Specific Hypoxia Data Derived from Quantitative Magnetic Resonance Imaging.* Cancers (Basel), 2021. **13**(19).
23. O'Connor, J.P.B., S.P. Robinson, and J.C. Waterton, *Imaging tumour hypoxia with oxygen-enhanced MRI and BOLD MRI.* Br J Radiol, 2019. **92**(1095): p. 20180642.
24. Werner, M. and H. Höfler, *Pathologie* in *Therapie gastrointestinaler Tumoren*, Jürgen D. Roder, Hubert J. Stein, and U. Fink, Editors. 2000, Springer Verlag: Berlin, Heidelberg. p. 51.
25. Becker, K., et al., *Histomorphology and grading of regression in gastric carcinoma treated with neoadjuvant chemotherapy.* Cancer, 2003. **98**(7): p. 1521-1530.
26. Thies, S. and R. Langer, *Tumor Regression Grading of Gastrointestinal Carcinomas after Neoadjuvant Treatment.* Frontiers in Oncology, 2013. **3**(262).
27. AIO, ACO, and ARO, *Konsentierte Stellungnahme der AIO, der ACO und der ARO zur neoadjuvanten Therapie beim Rektumkarzinom*. 2020, AIO: .
28. ARO, AIO, and ACO, *Konsentierte Stellungnahme der ACO, der AIO und der ARO zum „watch and wait"-Konzept mit intendiertem Organerhalt bei Rektumkarzinomen des mittleren und unteren Drittels*. 2020: https://www.aio-portal.de/stellungnahmen.html.
29. Marshall, J.B., et al., *Prospective evaluation of optimal number of biopsy specimens and brush cytology in the diagnosis of cancer of the colorectum.* Am J Gastroenterol, 1993. **88**(9): p. 1352-4.
30. Quirke, P., et al., *European guidelines for quality assurance in colorectal cancer screening and diagnosis. First Edition--Quality assurance in pathology in colorectal cancer screening and diagnosis.* Endoscopy, 2012. **44 Suppl 3**: p. Se116-30.
31. Elm, E.v., et al., *Strengthening the reporting of observational studies in epidemiology (STROBE) statement: guidelines for reporting observational studies.* BMJ, 2007. **335**(7624): p. 806-808.
32. Collins, G.S., et al., *Transparent reporting of a multivariable prediction model for individual prognosis or diagnosis (TRIPOD): the TRIPOD Statement.* BMC Medicine, 2015. **13**(1): p. 1.
33. Schulz, K.F., D.G. Altman, and D. Moher, *CONSORT 2010 Statement: updated guidelines for reporting parallel group randomised trials.* BMJ, 2010. **340**: p. c332.
34. Karnofsky, D.A., et al., *The use of the nitrogen mustards in the palliative treatment of carcinoma. With particular reference to bronchogenic carcinoma.* Cancer, 1948. **1**(4): p. 634-656.
