# Supplementary material for: Planning adaptive treatment by longitudinal response assessment implementing MR imaging, liquid biopsy and analysis of microenvironment during neoadjuvant treatment of rectal cancer (PRIMO)
Source: Medicine (Baltimore). 2023 Apr 28;102(17):e33575. doi: 10.1097/MD.0000000000033575 (PMC10146036; doi:10.1097/MD.0000000000033575)
Supplement: Supplementary file 2 [file medi-102-e33575-s002.pdf]

Enrolment and assessment schedule

All clinical visits (V1-V22) and scheduled assessment are shown. Primary Endpoints will be assessed by resection / histological sampling (OP). V9-V22 comprise two points of time, depending whether consolidating chemotherapy (“Total Neoadjuvant Therapy”, TNT) was performed or resection is scheduled after 8 weeks therapy-free interval.

Point of Time / Visit	**Screening**	**V0**	**V1**	**V2[3]**	**V3**	**V4**	**V5**	**V6**	**V7**	**V8**	**V9 / OP[1]**	**V10[2,3]**	**V11[2,3]**	**V12[2,3]**	**V13[2,3]**	**V14[2,3]**	**V15[2,3]**	**OP[2]**	**Tiered analysis: primary endpoint**
**Days** (since enrollment)	-42-0	1	2-14	15-28	29-35	36-49	50-56	64-70	78-84	92-98	106-112	120-126	134-140	141-147	162-168	176-182	218-224	232-238	
**Weeks** (since enrollment)	**-6-0**	**1**	**2-3**	**3-4**	**5**	**6-7**	**8**	**10**	**12**	**14**	**16**	**18**	**20**	**22**	**24**	**26**	**32**	**34**	
	**Procedures**																		
5-FU-/Oxaliplatin Chemo therapy (2x, q2w)				X		X													
Radiatiotherapy (28 fractions)				X	X	X	X												
TNT: FOLFOX4 Chemotherapy (9x, q2w)								X[2]	X[2]	X[2]	X[2]	X[2]	X[2]	X[2]	X[2]	X[2]			
Histological sampling (TNM): primary diagnosis	X																		
Resection / serial biopsies[2] (ypTNM / TRG-Score)											X[1]							X[2]	
	**Enrolment**																		
Eligibility screening		X																	
Informed consent		X																	
Demographic Data		X																	
	**Assessments**																		
**Multiparametric-MRI**			X		X		X			X			X[2]				X[2]		
**Measurement of TILs**	X											X[1]						X[2]	
**Measurement of CTCs**			X		X		X			X[1]/ X[2]	X[1, 4]						X[2]	X[2, 4]	
QoL Questionnaires (QLQ-C30, CR-29)		X					X												
Toxicity Questionnaires (CTCAE)		X			X		X			X			X[2]				X[2]		
KPS (%)		X		X	X	X	X	X	X	X	X[1] / X[2]	X[2]	X[2]	X[2]	X[1] / X[2]	X[2]			
CEA		X			X		X[1] / X[2]			X[1] / X[2]			X[2]				X[2]		
IL-6		X			X		X[1] / X[2]			X[1] / X[2]			X[2]				X[2]		
Routine blood samples	X			X	X	X		X[2]	X[2]	X[2]	X[2]	X[2]	X[2]	X[2]	X[2]	X[2]	X[2]		

Point of Time / Visit	**V16**	**V17**	**V18**	**V19**	**V20**	**V21**	**V22**	**Close out**
**Months** (since enrollment)	6[1] / 10,5[2]	10[1] / 14,5[2]	16[1] / 20,5[2]	28[1] / 32,5[2]	40[1] / 44,5[2]	52[1] / 56,5[2]	64[1] / 68,5[2]	
**Weeks** (since enrollment)	**24[1] / 42[2] (8 W. post-OP)**	**40[1] / 58[2] (6 Monate post-OP)**	**64[1] / 82[2] (12 Monate post-OP)**	**112[1] / 130[2] (24 Monate post-OP)**	**160[1] / 178[2] (36 Monate post-OP)**	**208[1] / 226[2] (48 Monate post-OP)**	**256[1] / 274[2] (60 Monate post-OP)**	
	**Procedures**							
**Follow-up**	X	X	X	X	X	X	X	
	**Assessments**							
**Multiparametric-MRI**								
**Measurement of TILs**								
**Measurement of CTCs**	X	X	X	X	X	X	X	
**Specific follow-up**	X	X	X	X	X	X	X	
QoL Questionnaires (QLQ-C30, CR-29)	X		X				X	
Toxicity Questionnaires (CTCAE)	X	X	X	X	X	X	X	
CEA	X	X	X	X	X	X	X	
Routine blood samples	X	X	X	X	X	X	X	

1 – *if patient does not receive consolidating chemotherapy (no TNT)*
2 – *if patient receives consolidating chemotherapy (TNT: consolidating FOLFOX4)*
3 –*Visits V10-V15 are only applicable if consolidating chemotherapy was conducted*[2]
4 – *Measurement of CTCs within 5 days after resection (OP + 0-5d)*

Abbreviations: *CEA – Carcinoembryonic Antigen, CTC – Circulating Tumor Cells, IL-6 – Interleukin 6, KPS – Karnofsky Performance Score, MRI – Magnetic Resonance Imaging, QoL – Quality of Life, TILs – Tumor-infiltrating Lymphocytes, TNT- Total Neoadjuvant Therapy, TRG – Tumor Regression Grading*
