# Supplementary material for: Planning adaptive treatment by longitudinal response assessment implementing MR imaging, liquid biopsy and analysis of microenvironment during neoadjuvant treatment of rectal cancer (PRIMO)
Source: Medicine (Baltimore). 2023 Apr 28;102(17):e33575. doi: 10.1097/MD.0000000000033575 (PMC10146036; doi:10.1097/MD.0000000000033575)
Supplement: Supplementary file 3 [file medi-102-e33575-s003.pdf]

Supplemental digital content 3

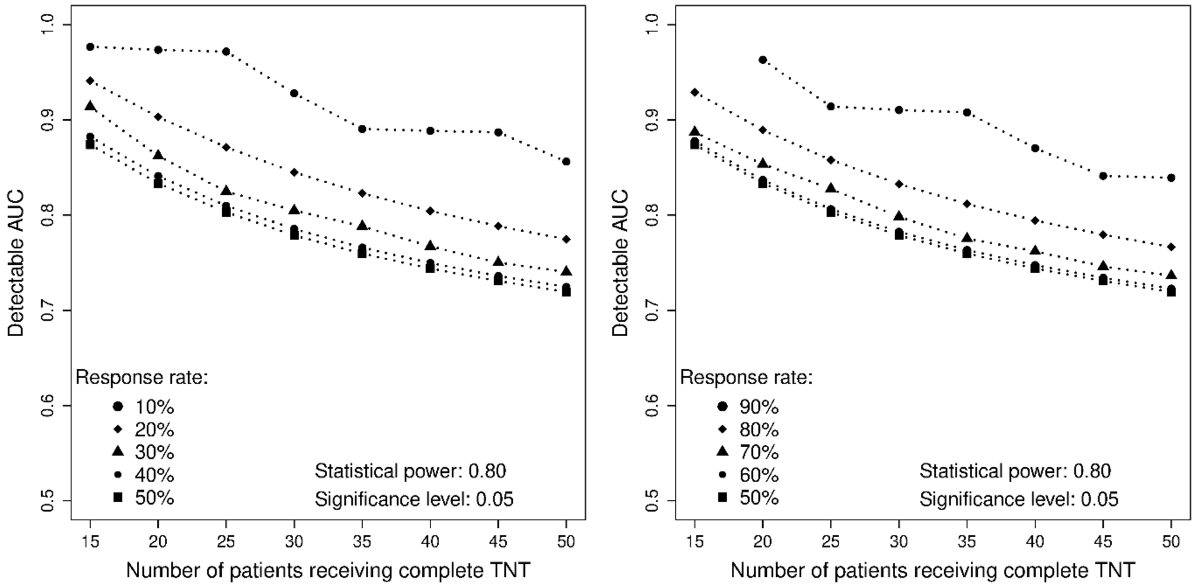

Figure 3. Detectable AUC depending on sample size (*here:* number of patients receiving complete TNT) and response rate (left panel: ≤50%; right panel: ≥50%) for given statistical power and significance level. Note: Detectable AUC could not be calculated for a response rate of 90% in a sample of 15 patients. Abbreviations: AUC, area under receiver operation characteristic (ROC) curve; TNT, total neoadjuvant therapy.
